# Structure and repair of replication-coupled DNA breaks

**DOI:** 10.1126/science.ado3867

**Published:** 2024-08-16

**Authors:** Raphael Pavani, Veenu Tripathi, Kyle B. Vrtis, Dali Zong, Raj Chari, Elsa Callen, Ajith V. Pankajam, Gang Zhen, Gabriel Matos-Rodrigues, Jiajie Yang, Shuheng Wu, Giordano Reginato, Wei Wu, Petr Cejka, Johannes C. Walter, André Nussenzweig

**Affiliations:** 1 Laboratory of Genome Integrity, National Cancer Institute, NIH, Bethesda, MD, USA.; 2 Department of Biological Chemistry and Molecular Pharmacology, Blavatnik Institute, Harvard Medical School, Boston, MA, USA.; 3 Genome Modification Core, Frederick National Lab for Cancer Research, Frederick, MD, USA.; 4 State Key Laboratory of Molecular Biology, Shanghai Institute of Biochemistry and Cell Biology, Center for Excellence in Molecular Cell Science, Chinese Academy of Sciences, Shanghai, China.; 5 Institute for Research in Biomedicine, Universita della Svizzera italiana (USI), Faculty of Biomedical Sciences, Bellinzona, Switzerland.; 6 Howard Hughes Medical Institute, Harvard University, Boston, MA, USA.

## Abstract

Using CRISPR-Cas9 nicking enzymes, we examined the interaction between the replication machinery and single-strand breaks, one of the most common forms of endogenous DNA damage. We show that replication fork collapse at leading-strand nicks generates resected single-ended double-strand breaks (seDSBs) that are repaired by homologous recombination (HR). If these seDSBs are not promptly repaired, arrival of adjacent forks creates double-ended DSBs (deDSBs), which could drive genomic scarring in HR-deficient cancers. deDSBs can also be generated directly when the replication fork bypasses lagging-strand nicks. Unlike deDSBs produced independently of replication, end resection at nick-induced seDSBs and deDSBs is BRCA1-independent. Nevertheless, BRCA1 antagonizes 53BP1 suppression of RAD51 filament formation. These results highlight distinctive mechanisms that maintain replication fork stability.

## Abstract (summary)

**INTRODUCTION:**

Homologous recombination (HR) promotes error-free repair of double-strand breaks (DSBs). Mutations in HR genes, such as *BRCA1* and *BRCA2*, predispose individuals to breast, ovarian, and pancreatic cancers. Studies of HR-deficient tumors have unveiled a distinct mutational signature, characterized by small insertions, deletions, and rearrangements dispersed throughout the genome. This pattern highlights the existence of endogenous sources of DSBs that, in the absence of HR, are repaired by mutagenic end-joining pathways. Because HR-deficient cancer cells are hypersensitive to chemotherapeutic drugs that induce DSBs during replication, such as inhibitors of poly (adenosine diphosphate–ribose) polymerase (PARPi) and DNA topoisomerase 1 (TOP1i), the relevant initial lesions are likely to be single-strand breaks (SSBs) that are converted into DSBs by replication fork passage.

**RATIONALE:**

Although most proteins critical for DSB repair have been identified, their cellular functions have been largely studied outside the context of replication and using agents such as ionizing radiation or endonucleases that cut both strands of DNA independently of replication. However, unlike canonical replication-independent DSBs, which have two clear ends (i.e., double-ended DSBs, or deDSBs), replication-coupled DSBs have been assumed to be single-ended (i.e., seDSBs). We therefore hypothesized that replication-coupled DSBs may elicit distinct cellular responses. To test this idea, we devised a Cas9 nickase (nCas9) system to synchronously introduce site-specific DNA nicks (i.e., SSBs) into the human genome in a controlled manner. Leveraging next-generation sequencing and cutting-edge molecular imaging technologies, we systematically explored the consequences of the collision of these nicks with replication forks. To study whether the location of the nick (on the leading or lagging strand) could influence replication-coupled DSB generation and/or repair, we selected regions with unidirectional replication forks. We also studied the function of BRCA1 and 53BP1 at replication-coupled DSBs, given their well-established antagonistic role in the repair of canonical DSBs.

**RESULTS:**

We found that a fork encountering a leading-strand nick produces a seDSB, consistent with the replisome “running off” at the nick site. Notably, collision with a lagging strand nick can produce a deDSB under certain conditions, as when the replication fork bypasses the nick and continues DNA synthesis. In all cases, the resultant DSBs are repaired by RAD51-mediated HR. If the leading-strand seDSBs are not resolved quickly, the eventual arrival of a converging fork can convert them into deDSBs, which in turn can serve as substrates for mutagenic end joining, leading to characteristic genomic alterations observed in HR-deficient cancers.

5′–3′ resection is an early key step in HR that is believed to be controlled by BRCA1 and 53BP1 proteins. Studies using endonucleases and irradiation have concluded that BRCA1 stimulates end resection at replication-independent DSBs, whereas 53BP1 and its effectors counteract end resection through fill-in synthesis. In addition to enabling end resection, BRCA1 also stimulates HR by loading RAD51 onto single-strand DNA to initiate strand invasion. We show that, whereas BRCA1 facilitates end resection at deDSBs induced outside of S phase, resection at nick-induced DSBs increases in BRCA1-deficient cells. This is consistent with the finding that mutagenic DNA repair junctions induced by collapsed replication forks exhibit longer deletions and increased microhomology usage compared with wild-type cells. A lack of involvement for BRCA1 in end resection marks a major difference that distinguishes replication-coupled DSBs from canonical DSBs. Nevertheless, BRCA1 remains essential for HR-mediated repair of replication-coupled DSBs because it is required to overcome 53BP1 suppression of RAD51 filament formation.

**CONCLUSION:**

Our work provides a comprehensive high-resolution view of how replication-coupled DSBs are generated, processed, and repaired. In addition to revealing an unexpected asymmetry between leading- and lagging-strand fork collapse, we uncovered major differences in the processing of replication-coupled versus canonical DSBs. These results highlight distinctive mechanisms that operate in our cells to maintain replication fork stability.

## Graphical Abstract

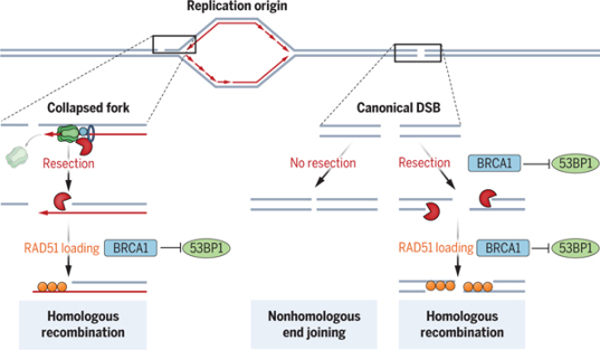

**Canonical and replication-associated DSBs are processed differently**. (Right) At canonical DSBs, the end-resection machinery along with other DNA damage response proteins (e.g., BRCA1 and 53BP1) are recruited to lesions de novo. Here, the pro- and anti-resection activities of BRCA1 and 53BP1 directly compete to regulate resection and the choice between nonhomologous end joining and HR. (Left) Resection nucleases travel with unperturbed replication forks and commit cells to HR upon fork collapse. BRCA1 acts downstream of resection to limit 53BP1’s inhibitory action on loading RAD51 to resected ends.

The replication fork stalls when it encounters obstacles such as noncanonical secondary DNA structures, protein-DNA complexes, or damage in the template (1, 2). A common response to replication stress is fork reversal, whereby adenosine triphosphate–dependent translocases including HLTF, SMARCAL1, and ZRANB3 promote displacement and annealing of nascent strands. BRCA1, BRCA2, RAD51, and other homologous recombination (HR) proteins stabilize andshield reversed forks from nuclease-mediated degradation, setting the stage for fork restart (3). During this process, the replication machinery, including the CMG helicase, remains bound to the stalled fork (4). However, when a fork breaks and/or the replisome is disassembled (hereafter referred to as fork collapse), it can instead create a double-strand break (DSB) that requires HR to resume replication and maintain genome stability (5, 6). Fork collapse occurs spontaneously in every S phase (7) and is induced by chemotherapeutic agents such as poly(adenosine diphosphate–ribose) polymerase inhibitors (PARPis), topoisomerase inhibitors, and DNA cross-linking agents, which underpin their efficacy against fast-proliferating cancer cells, especially those with HR deficiency (8).

Most cell-based systems designed to study HR have used endonucleases that generate DSBs independently of replication (9). These studies have identified 5′–3′ end resection as an early key step in HR (10), a process that is controlled by BRCA1 and 53BP1 proteins (11). BRCA1 promotes end resection at replication-independent (canonical) DSBs (12, 13), whereas 53BP1 and its effectors (RIF1, Shieldin, CST, polymerase α–primase) counteract resection through fill-in DNA synthesis (14, 15), thereby limiting single-stranded DNA (ssDNA) generation and favoring nonhomologous end joining (NHEJ). In addition to enabling end resection, BRCA1 also directly stimulates recombination by facilitating PALB2/BRCA2-dependent loading of RAD51 recombinase onto replication protein A (RPA)–coated ssDNA to initiate strand invasion (16). Thus, loss of BRCA1 is thought to impair both resection and recombination.

Unlike canonical DSBs that have two clear ends (i.e., deDSB), replication-coupled DSBs are assumed to be single-ended (i.e., seDSB). This potential difference in end structure may account for some apparent “discrepancies” in the processing of replication-coupled versus canonical DSBs. Most notably, whereas HR deficiency clearly skews repair of canonical deDSBs toward error-prone NHEJ (16), the latter does not appear to compete with HR to resolve DNA nicks during replication (17). Yet loss of 53BP1 rescues the embryonic lethality of BRCA1-deficient mice and confers resistance to chemotherapies that challenge the replication fork such as PARPi and camptothecin (CPT) (18, 19). This raises the question as to whether BRCA1’s role in promoting resection is limited to canonical deDSBs and hints at the existence of a critical BRCA1–53BP1 control point downstream of end resection, perhaps at the level of RAD51 filament formation and/or subsequent strand invasion (12, 20, 21).

Recent studies attempting to model DNA repair in the context of replication have yielded somewhat contradictory conclusions as to whether a nick needs to be converted to a DSB to trigger HR (17, 22, 23). It was also noted that replication fork collision with a nick on the lagging strand appears to stimulate HR more strongly (17) and is more dependent on BRCA1 for repair accuracy (22) than a leading-strand collapse. However, as these investigations only examined the final repair product of fork collapse, the mechanisms underlying such strand asymmetries remain unexplained. Here, we combine direct analyses of DNA end structures at sites of nick-induced fork collapse with detailed mapping of repair factor binding to establish a comprehensive high-resolution view of how replication-coupled DSBs are generated, processed, and repaired.

## A system to analyze DNA end structures at sites of nCas9-induced fork collapse

**Fig. 1.**
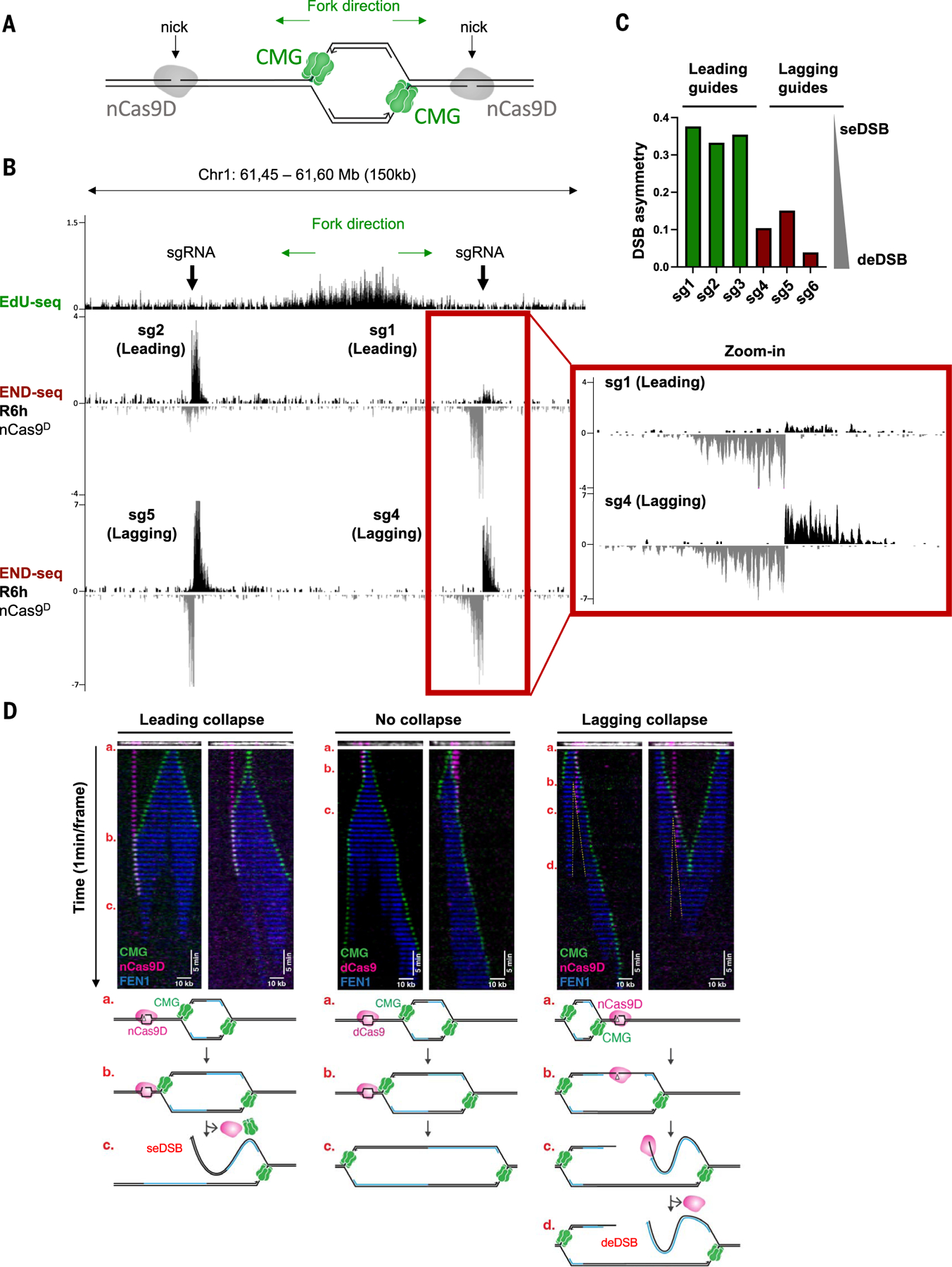
Distinct DNA end structures are generated by fork collision with leading-strand versus lagging-strand nicks. **(A)** Schematic showing origin firing, Cas9 nickase D10A (nCas9^D^), and the CMG helicase. **(B)** Genome browser screenshots displaying EdU-seq and END-seq profiles as normalized read density [reads per million (RPM)] in MCF10A cells. Top panels depict the position of individual nicking sgRNAs with respect to the targeted replication initiation zones, as mapped by EdU-seq in MCF10A cells released from G_1_ arrest in the presence of 4 μM APH. We chose relatively isolated origins with strong fork unidirectionality: Forks converging from the left and right side of the targeted replication origin shown in (B) are predicted to be ~1200 and ~350 kb away, respectively. Lower panels show END-seq signals generated by Cas9 D10A nickase (nCas^D^) at leading-strand (middle) or lagging-strand (bottom) fork collapse in MCF10A cells 6 hours after release from G_1_ arrest (R6h). Doxycycline was added during G_1_ arrest to induce nCas^D^ expression. Positive- and negative-strand END-seq reads are displayed in black and gray, respectively. Green arrows show replication fork direction. **(C)** Asymmetry at leading-strand versus lagging-strand nick-induced DSBs. The degree of DSB asymmetry was calculated as described in the Materials and methods. Values closer to 0 denote more symmetrical breaks (deDSB). **(D)** Single-molecule imaging of replication fork collapse. Tethered DNA was nicked in the leading or lagging strand with nCas9^D-Atto550^ or incubated with dCas9^Atto550^ and replicated in *Xenopus* extracts containing Fen1^mKikGR^ and GINS^AF647^. CMG is shown in green, Fen1mKikGR in blue, and nCas9^D^ or dCas9 in magenta. Kymographs were created by stacking the frames of a movie (1-min intervals) and show representative molecules from two independent biological replicates. For leading-strand collapse (left panel), a total of 89 molecules were quantified, and CMG was lost at the break site in 70% of the events (63/89). For lagging-strand collapse (right panel), a total of 58 molecules were quantified, and CMG bypasses nCas9 in 86% of the events (50/58). For “no collapse” (dCas9) (middle panel), CMG helicase bypasses dCas9 in 97% of the molecules (57/59), which dissociates from DNA soon after CMG collision. Top panels above each kymograph show tethered DNA (white) and nCas9 (magenta) imaged before egg extract addition. Schematic representation of different replication fork–nCas9 collision outcomes are shown at the bottom.

To study fork collapse in human cells, we first mapped replication initiation zones by EdU (5-ethynyl-2′-deoxyuridine) sequencing (EdU-seq) (24) in cells synchronously entering S phase in the presence of aphidicolin (APH; fig. S1A). The locations of early replication origins detected by EdU-seq were similar to those derived from Okazaki fragment sequencing [OK-seq (25)] or transferase-activated end ligation sequencing [TrAEL-seq (26)] (fig. S1A). To ensure collision with unidirectional replication forks, we targeted individual Cas9 nickase (nCas9) nicks adjacent to relatively isolated origins situated at least 350 kb from distal initiation zones (Fig. 1, A and B, and fig. S1A). Two doxycycline-inducible Cas9 nickases were used: HNH-mutant H840A (nCas9^H^) and RuvC-mutant D10A (nCas9^D^) (fig. S1B), which nick the nontarget and target strands, respectively (fig. S1C). Nicking by nCas9^H^ releases the non-target strand, making its 3′ end accessible to the damage surveillance machinery (27). In contrast, nCas9^D^ remains tightly associated with the target strand and single-guide RNA (sgRNA) after cleavage (fig. S1C) (27).

To initiate nicking, nCas9^H^ or nCas9^D^ were individually induced for 20 hours in G_1_-arrested MCF10A cells before synchronous release into S phase (fig. S1D). At different time points after release, we determined the DNA end structure by END-seq (28). A single dominant END-seq peak (an asymmetrical end) is indicative of a seDSB, whereas a deDSB would produce two symmetrical DNA ends. We verified that no DSBs were detectable in G_1_-arrested cells expressing nCas9^D^ (fig. S1F) or in cycling cells expressing a catalytically dead Cas9 (dCas9) (fig. S1, E and F).

## HLTF prevents nCas9^H^ from inducing replication fork collapse

In vitro single-molecule imaging of frog egg extracts has revealed that an nCas9^H^-induced nick stops replication and causes the CMG helicase to rapidly unload, suggestive of fork collapse (6). However, we detected little or no END-seq signal after replication fork collision with an nCas9^H^-induced nick in intact human cells (fig. S2, A to C).

Free 3′ DNA ends are recognized and bound by the HLTF translocase (29). As DNA nicks produced by nCas9^H^ have accessible 3′ ends, we hypothesized that HLTF is recruited to these lesions and potentially dislodges the nCas9^H^ using its motor activity. In this scenario, the removal of nCas9^H^ by HLTF might promote nick repair before replication fork arrival, thereby reducing the number of collision events. Consistent with this, we observed an increase in END-seq signal at sites of both leading- and lagging-strand nCas9^H^ nicks when HLTF was absent (fig. S2, A to C). The END-seq peaks were asymmetrical and spread from the nick site, which in theory could arise from DNA ends generated by fork collapse or fork reversal (2). However, as HLTF is known to promote fork reversal (30), the observed END-seq signal in HLTF-deficient cells is more consistent with resected seDSBs. We propose that active removal of nCas9^H^ by HLTF explains the more limited mutagenic potential and the decreased HR stimulation by nCas9^H^ compared with nCas9^D^ (17, 31) as well as improved nCas9^H^-mediated prime editing in cells lacking HLTF (32).

## nCas9^D^ generates distinct DNA end structures at leading- and lagging-strand collapse

Unlike nCas9^H^, END-seq peaks were readily identified in replicating cells expressing nCas9^D^ independently of whether HLTF was expressed (Fig. 1, A to C, and figs. S2D and S3A). DSBs were highly asymmetrical at sites of nCas9^D^-induced leading strand collapse (DSB asymmetry: 0.3 to 0.4; Fig. 1, B and C, and fig. S3A). By contrast, DNA ends at nCas9^D^-induced lagging collapse were more symmetrical (DSB asymmetry: 0 to 0.1; Fig. 1, B and C, and fig. S3A), resembling the end structure observed at canonical deDSBs, such as those generated by wild-type (WT) Cas9 (see below) or restriction enzymes (28). We conclude that nicks created by nCas9^D^ lead to one- or two-ended breaks after collision with a replication fork, depending on whether the nick is targeted to the leading or lagging strand, respectively.

## deDSB formation at lagging-strand collapse occurs independently of converging forks

Although we targeted relatively isolated origins, it is possible that adjacent forks from the opposite direction could eventually collide with the nick, giving rise to a deDSB (fig. S3B). We therefore impeded distal replication forks by using two additional “blocking” sgRNAs (fig. S4A). All three sgRNAs generated DNA nicks in G_1_-arrested cells that were sensitive to S1 nuclease digestion [S1-END-seq (33) with relative intensity: sgBlock1 > sg4 > sgBlock2] (fig. S4A). By quantitative polymerase chain reaction (qPCR), we estimated that sg4 and sgBlock2 generated nicks in at least 50 and 55% of cells, respectively (fig. S4B). When cells were released intoS phase, we observed DSBs at the positions of the two blocking sgRNAs (sgBlock2 > sgBlock1), indicating that leftward moving forks were stopped by the blocking nicks (fig. S4A). The fact that the outermost nick (sgBlock2) induced more breaks than did the proximal nick (sgBlock1) suggests that fork stalling at this blocking site was efficient. The symmetrical deDSB structure originating from rightward moving forks remained unchanged in the presence of sgBlock1 and sgBlock2 (fig. S4A). These results suggest that lagging-strand fork collapse generates deDSBs independently of converging forks.

## deDSB formation at lagging-strand collapse occurs by replication fork bypass

If converging forks do not account for the second DNA end during lagging-strand fork collapse, we considered the possibility that deDSBs may result from replication bypassing the nick (fig. S4C). To test this idea, we used single-molecule imaging to monitor fork collapse in real time in frog egg extracts (6). We simultaneously imaged the nCas9^D^ complex (labeled with Atto550), recombinant GINS (a subunit of CMG helicase labeled with Alexa Fluor 647), and photoactivatable FEN1 (Fen1^mKikGR^), which binds proliferating cell nuclear antigen on nascent DNA (6). Upon collision with a leading-strand nick generated by nCas9^D^, CMG stalled for a few minutes, and then CMG and nCas9^D^ disappeared simultaneously (Fig. 1D, leading collapse). These observations support a model in which a seDSB is generated when the replisome runs off the broken leading-strand template (6). Notably, when CMG encountered dCas9, CMG temporarily stalled and then resumed progression upon dCas9 dissociation (Fig. 1D, no collapse) (6), confirming that a Cas9 protein block per se does not induce fork collapse.

The behavior of CMG at an nCas9^D^-induced nick in the lagging-strand template was markedly different. In this case, nCas9^D^ was not displaced by CMG; rather, CMG bypassed nCas9^D^ 86% of the time, with the FEN1 nascent DNA signal trailing the CMG helicase (Fig. 1D, lag collapse). This indicates that nCas9^D^ remained engaged with the target strand while the replisome passed by, to continue synthesizing DNA beyond the nick. We envisage that a deDSB is generated when nCas9^D^ dissociates from DNA and Okazaki fragment synthesis terminates at or near the nick, which is then subjected to 5′–3′ end resection (Fig. 1D and fig. S4C, lagging collapse). Indeed, we observed a gap in FEN1 signal emanating from the nick site (Fig. 1D, lagging collapse, yellow dashed line). This model is therefore consistent with the resected deDSB end structure we observed in human cells when a replication fork collides with a nCas9^D^-induced nick on the lagging strand (Fig. 1B and fig. S3A).

## Resolution of nick-induced DSBs is dependent on RAD51

To determine the fate of DNA ends produced by leading- and lagging-strand nicks, we collected MCF10A cells at different time points after G_1_ release (fig. S5A). At 3 hours after release, nCas9^D^-induced DSBs became detectable and were already resected (fig. S5, B and C). DSBs increased at 6 hours, likely reflecting a greater number of cells in which the fork reaches the nick sites. DSBs then largely disappeared by 12 hours (fig. S5, B to D). Maximum resection tract lengths (~5 kb) were similar for leading- and lagging-strand nick-induced DSBs (fig. S5E) but were four- to fivefold greater than those previously reported in yeast (34).

**Fig. 2.**
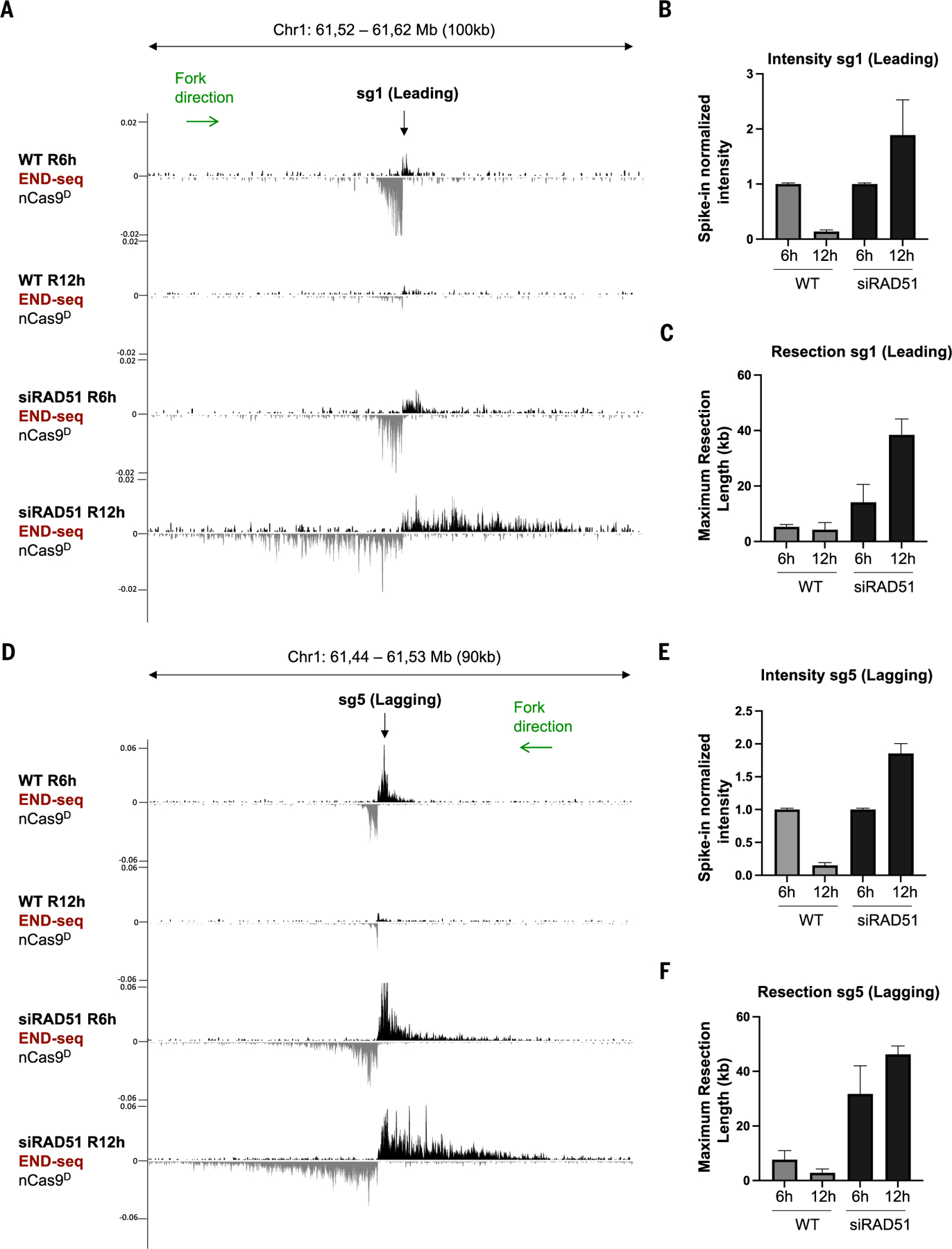
Resolution of collapsed forks is dependent on RAD51. **(A)** Genome browser screenshots displaying END-seq signals at a leading-strand fork collapse generated by nCas9^D^/sg1 in MCF10A cells treated with siRAD51 or untreated. Cells were collected 6 hours (R6h) or 12 hours (R12h) after release from G_1_ arrest. **(B)** Spike-in normalized END-seq intensity signal at sg1 collapsed forks quantified from three independent replicates. Intensity at the R6h time point was normalized to 1, with signals at R12h calculated relative to R6h. **(C)** Maximum resection tract lengths at nCas9^D^/sg1-induced DSB quantified from three independent replicates. **(D)** Genome browser screenshots displaying END-seq signals at a lagging-strand nick-induced DSB generated by nCas9^D^/sg5 in MCF10A cells treated with siRAD51 or untreated. Cells were collected 6 hours (R6h) or 12 hours (R12h) after release from G_1_ arrest. Positive- and negative-strand END-seq reads in (A) and (D) are displayed in black and gray, respectively. Green arrows show replication fork direction. **(E)** Normalized END-seq intensity at nCas9^D^/sg5-induced DSB quantified from three independent replicates. **(F)** Maximum resection tract lengths quantified from three independent replicates. Error bars in (B), (C), (E), and (F) represent standard deviation.

The repair of collapsed forks has been previously reported to be dependent on RAD51 (5) and on the break-induced replication (BIR) factors RAD52 (35) and POLD3 (36). When we depleted RAD51, DSBs accumulated over time (Fig. 2 and fig. S6). Notably, maximum resection tract lengths increased from ~5 kb to >40 kb, indicating hyper-resection for both leading- and lagging-strand nick-induced DSBs in the absence of RAD51 (Fig. 2, C and F, and fig. S6).

To deplete RAD52 and POLD3, we used RPE-1 cells because of greater knockdown efficiencies of siRAD52 and siPOLD3 in this cell line. RPE-1 cells exit G_1_ arrest somewhat more slowly than MCF10A (fig. S7A); as such, we found that nick-induced DSBs were readily detectable by 8 hours after release and were subsequently resolved by 16 hours (fig. S7B). Knockdown of POLD3 or RAD52 had no effect on DSB resolution (fig. S7, B to D). Thus, the repair of nick-induced DSBs requires RAD51 but does not depend on genes implicated in BIR, consistent with studies in yeast demonstrating RAD51-dependent HR but not BIR as the primary repair pathway at broken replication forks (5).

## seDSBs are converted to deDSBs in the absence of strand invasion

**Fig. 3.**
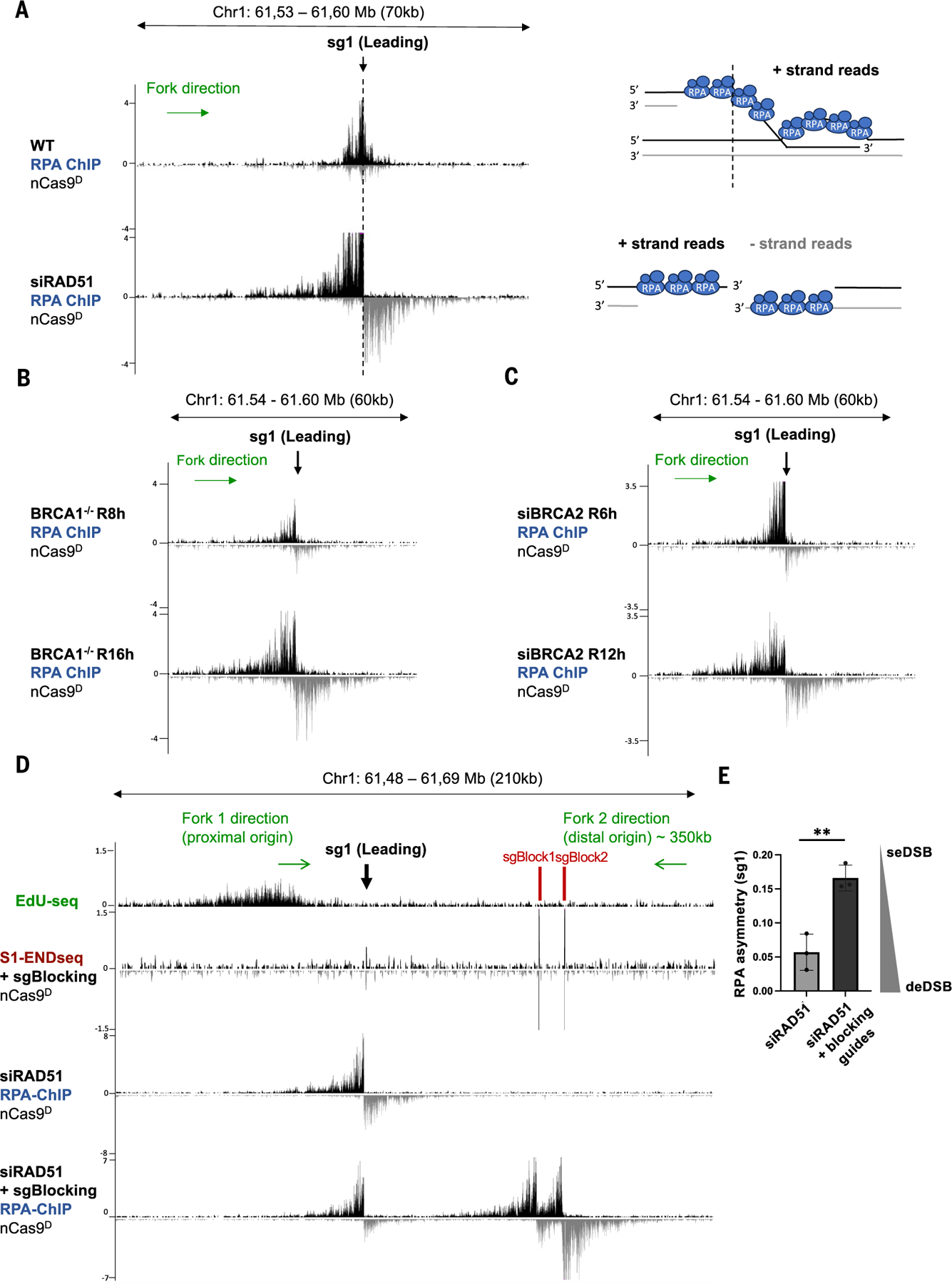
Generation of deDSBs at leading-strand collapse in the absence of RAD51. **(A)** Genome browser screen-shots displaying normalized RPA-bound ssDNA ChIP-seq signals (RPM) at a leading-strand fork collapse generated by nCas9^D^/sg1 in MCF10A cells treated with siRAD51 or untreated. Dashed line indicates the position of sg1. Schematic representations of RPA binding in WT and RAD51-depleted cells are shown on the right. In WT, RPA is bound to the resected strand (left side of dashed line) of a seDSB and potentially also to the exposed ssDNA in the migrating D-loop (right side of dashed line) after strand invasion. In RAD51-depleted cells, RPA is instead bound to resected ends on both sides of a deDSB. **(B)** Genome browser screen-shots displaying RPA ChIP-seq signals at a leading-strand fork collapse generated by nCas9^D^/sg1 in *BRCA1*^−/−^ RPE-1 cells released for 8 and 16 hours after G_1_ arrest. **(C)** Genome browser screenshots displaying RPA ChIP-seq at a leading-strand fork collapse generated by nCas9^D^/sg1 in BRCA2-depleted MCF10A cells released for 6 and 12 hours after G_1_ arrest. **(D)** Genome browser screen-shots displaying EdU-seq, S1-END-seq, and RPA-bound ssDNA ChIP-seq profiles. Top panel depicts the positions of the nicking and blocking sgRNAs with respect to the targeted replication initiation zone (fork 1 corresponds to the proximal origin) as well as the expected location of the converging fork (fork 2 corresponds to distal origin). Second panel from the top shows S1-END-seq in G_1_-arrested cells confirming successful expression and activities of nCas9^D^, sg1, sgBlock1, and sgBlock2. Third panel from the top and bottom panel show RPA-bound ssDNA ChIP-seq signals generated by nCas9^D^/sg1 in MCF10A in the absence or presence of blocking sgRNAs 6 hours after release from G_1_. Positive- and negative-strand RPA-bound ssDNA ChIP-seq and S1-END-seq reads in (A) to (D) are displayed in black and gray, respectively. **(E)** RPA asymmetry calculated as the ratio of positive/negative strand RPA reads from three independent replicates. Data are represented as mean ± standard deviation, and statistical significance is calculated using unpaired *t* test (**P < 0.005).

We noticed that highly resected deDSBs emerged in RAD51-depleted cells during nCas9^D^-induced leading strand collapse, distinct from seDSBs in WT cells (Fig. 2A). To further examine the structure of DNA at leading-strand collapse, we performed RPA ssDNA sequencing (37). In WT cells, collision of a rightward fork with the nick produced a pattern of RPA accumulation consistent with a 3′ resected seDSB (i.e., primarily positive strand signal) on the left side of the nCas9^D^ target site (Fig. 3A, dashed line denotes the location of the nick). We observe that a small but reproducible RPA signal extended to the right of the nick site (Fig. 3A), potentially indicative of ssDNA generated during strand invasion and displacement loop (D-loop) migration (38). In RAD51-deficient cells lacking strand invasion, this signal disappeared; instead, RPA bound strongly to 3′ ends with the opposite polarity, indicating the emergence of a highly resected 3′ ssDNA on the right side of the break (i.e., a deDSB) (Fig. 3A). Similar observations were made when leading-strand fork collapse was initiated from a leftward moving fork (fig. S8A). deDSBs also formed with leading-strand collapse in cells lacking BRCA1 or BRCA2 (Fig. 3, B and C). Notably, amplicon sequencing revealed high levels of deletions with microhomologies surrounding the nick sites in BRCA1-deficient cells (fig. S8, B to D), suggesting that RAD51-dependent strand invasion prevents the conversion of seDSBs to deDSBs, thereby avoiding potentially mutagenic repair.

We hypothesized that in the absence of functional HR, deDSBs produced from leading strand collapse might arise from adjacent replication forks. Upon fork runoff at a leading strand nick, a gap is created between the nascent Okazaki fragments and the parental lagging strand template (6, 39). If this “hybrid” gap is not sealed, a second DSB end could be generated by a converging fork (fig. S9). To test this idea, we impeded converging forks with two blocking sgRNAs (as in fig. S4A). The outermost blocking sgRNA (sgBlock2) generated nicks in 67% of cells, as measured by qPCR (fig. S10A). Efficient blocking of converging forks was evidenced by the presence of prominent S1-END-seq and RPA chromatin immunoprecipitation (ChIP) peaks at blocking sites (Fig. 3D). Genome-wide analysis of DNA replication using TrAEL-seq (26) also revealed that converging forks terminated prematurely at the blocking sites (fig. S10B). Blocking converging forks in RAD51-depleted cells decreased the formation of the second DSB end, resulting in increased RPA asymmetry (Fig. 3, D and E). Thus, unrepaired seDSBs at sites of leading-strand collapse are turned into deDSBs by the converging fork. The extent of RPA binding beyond the nick site, which was partly dependent on POLD3, increased upon fork blockage in WT cells (fig. S10,C and D), suggesting that converging forks could potentially limit DNA synthesis and D-loop migration.

We also considered the possibility that the second DSB end detected at sites of leading-strand fork collapse in HR-deficient cells arises from re-nicking of DNA by nCas9^D^ if the “hybrid” nick is sealed before arrival of the converging fork, as proposed by Diffley and colleagues (39). To prevent recutting after initial collision, we engineered a FKBP degron domain into nCas9^D^, allowing for induced acute proteasomal degradation by a small molecule. As nCas9^D^-FKBP was degraded within 30 min of dTAG-V1 treatment (fig. S11A), we added dTAG-V1 5 hours after G_1_ release to maximize fork collapse while minimizing recutting (fig. S11, B and C). Nevertheless, deDSBs emerged to the same extent in BRCA2-deficient cells whether or not nCas9^D^ was present after the initial cleavage (fig. S11D). Altogether, these results suggest that in the absence of HR-mediated strand invasion, converging forks collapse at the unsealed “hybrid” gap, thereby converting replication-coupled seDSBs to deDSBs (fig. S9).

## BRCA1 is dispensable for resection at collapsed replication forks

**Fig. 4.**
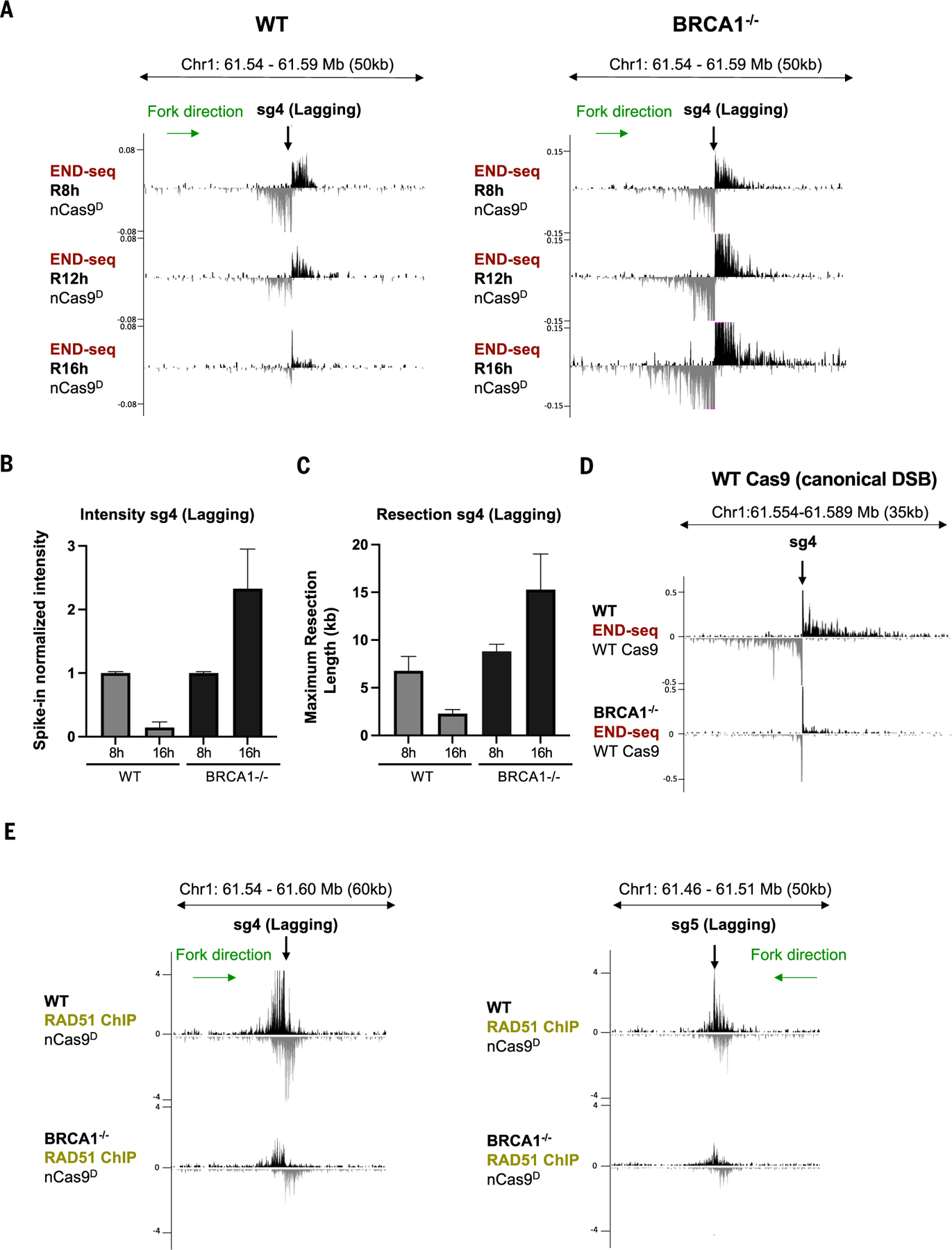
Resection at collapsed forks is BRCA1 independent. **(A)** Genome browser screenshots displaying END-seq signals at a lagging-strand nick-induced DSB generated by nCas9^D^/sg4 in WT and *BRCA1*^−/−^ RPE-1 cells. Cells were collected 8 hours (R8h), 12 hours (R12h), and 16 hours (R16h) after release from G_1_ arrest. **(B)** Spike-in normalized END-seq intensity signal at nCas9^D^/sg4-induced DSB quantified from three independent replicates. Intensity at the R8h time point was normalized to 1, with the signal at R16h calculated relative to R8h. **(C)** Maximum resection tract lengths at nCas9^D^/sg4-induced DSB quantified from three independent replicates. **(D)** Genome browser screenshots displaying END-seq signals at a canonical DSB generated by Cas9/sg4 in WT and *BRCA1*^−/−^ RPE-1 cells. **(E)** Genome browser screenshots displaying normalized RAD51 ChIP-seq signals at a lagging-strand nick-induced DSB generated by nCas9^D^/sg4 (left) and nCas9^D^/sg5 (right) in WT and *BRCA1*^−/−^ RPE-1 cells released for 8 hours after G_1_ arrest (*n* = 3 independent replicates). Positive- and negative-strand END-seq reads in (A) and (D) and RAD51 ChIP-seq reads in (E) are displayed in black and gray, respectively. Green arrows show replication fork direction. Error bars in (B) and (C) represent standard deviation.

BRCA1 is thought to facilitate recombination of canonical DSBs at two distinct steps: by promoting end resection (12, 13, 40, 41) and, subsequently, RAD51 filament formation and strand invasion (42–44). To determine whether BRCA1 fulfills a similar role in the repair of nick-induced DSBs, we examined DNA end structures at nCas9^D^-induced fork collapse in *BRCA1*^−/−^ RPE-1 cells (fig. S12A), which, as expected, were hypersensitive to replication stress triggered by PARPi treatment (fig. S12B). Notably, both leading- and lagging-strand nick-induced DSBs persisted in *BRCA1*^−/−^ cells for at least 16 hours after release, whereas in WT cells, maximum DSB intensity was reached by 8 hours and subsequently resolved by 16 hours (Fig. 4, A and B, and fig. S12, C and D). We found that resection tracts in *BRCA1*^−/−^ cells were even greater than WT cells at all time points (Fig. 4, A and C, and fig. S12, C and E). In contrast, when we coupled the same sgRNAs to WT Cas9, *BRCA1*^−/−^ cells clearly exhibited defective end resection at canonical deDSBs (Fig. 4D and fig. S12, F and G).

To substantiate these findings using a different system, we compared replication-dependent DSBs triggered by CPT and canonical DSBs generated by γ-irradiation (IR) in BRCA1-mutated MDA-MB-436 cells reconstituted with either empty vector (BRCA1^mut^) or WT BRCA1 cDNA (BRCA1^WT^) (45). Because DSBs in these cases are not site-specific, we monitored end resection and strand invasion by immunofluorescence detection of RPA and RAD51 foci, respectively. In response to IR, BRCA1^mut^ cells exhibited severely reduced RPA and RAD51 foci formation (fig. S13A). However, RPA foci formation was comparable in CPT-treated BRCA1^mut^ and BRCA1^WT^ cells, even though BRCA1 deficiency clearly impaired RAD51 loading (fig. S13B). Unlike BRCA1, we found that CtIP was required for resection at collapsed forks, induced either by CPT or by site-specific nicks (fig. S13, C to E). Thus, whereas CtIP is generally required for resection, BRCA1’s role in end processing appears to depend on the context in which DSBs are generated.

## 53BP1 counteracts RAD51 filament formation at nick-induced DSBs

Given that resection of DSBs after fork collapse remained intact, the inability of BRCA1-deficient cells to promptly repair these breaks might be due to a defect in RAD51 loading. Indeed, by chromatin immunoprecipitation sequencing (ChIP-seq), we detected a clear reduction in RAD51 binding at sites of nick-induced DSBs in *BRCA1*^−/−^ cells (Fig. 4E). Thus, the defect in resolving DNA ends at collapsed forks and the concomitant hyper-resection in *BRCA1*^−/−^ cells correlates with its essential downstream role in loading RAD51.

**Fig. 5.**
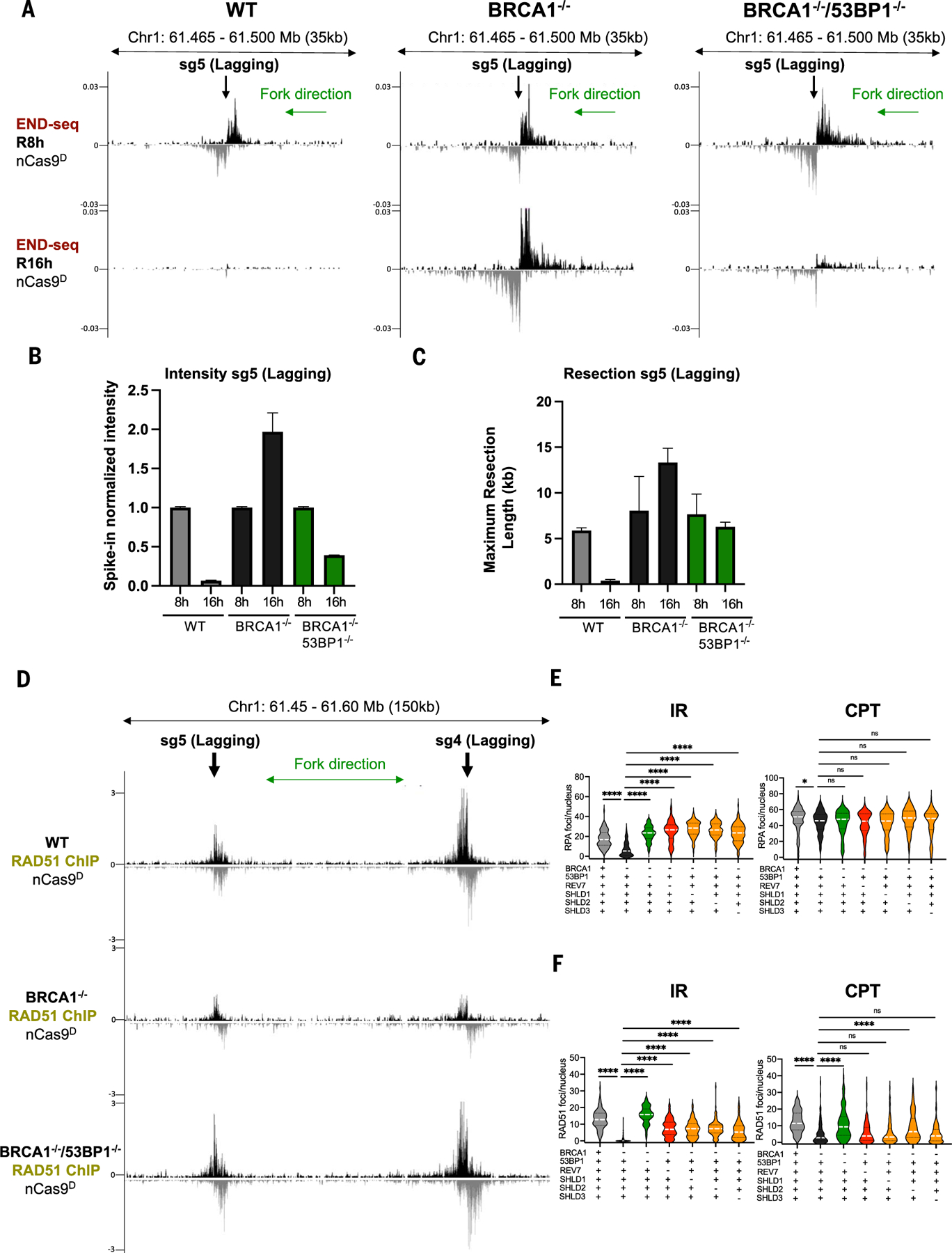
53BP1 inhibits RAD51 loading at collapsed forks. **(A)** Genome browser screenshots displaying END-seq signals at a lagging strand nick-induced DSB generated by nCas9^D^/sg5 in WT, *BRCA1*^−/−^, and *BRCA1*^−/−^*53BP1*^−/−^ RPE-1 cells. Cells were collected 8 hours (R8h) and 16 hours (R16h) after release from G_1_ arrest. **(B)** Spike-in normalized END-seq signal intensity at nCas9^D^/sg5-induced DSB quantified from two independent replicates. Intensity at the R8h time point was normalized to 1, with the signal at R16h calculated relative to R8h. **(C)** Maximum resection tract lengths quantified from two independent replicates. **(D)** Genome browser screenshots displaying normalized RAD51 ChIP-seq signals at lagging strand nick-induced DSB generated by nCas9^D^/sg4 and nCas9^D^/sg5 in WT, *BRCA1*^−/−^, and *BRCA1*^−/−^*53BP1*^−/−^ RPE-1 cells (*n* = 3 independent replicates). Positive- and negative-strand END-seq and RAD51 ChIP-seq reads in (A) and (D) are displayed in black and gray, respectively. Green arrows show replication fork direction. (E) RPA foci in EdU positive nuclei measured 3 hours after 5 Gy IR or 1 hour after 1 μM CPT treatment in WT, *BRCA1*^−/−^, *BRCA1*^−/−^*53BP1*^−/−^, *BRCA1*^−/−^ *SHLD1*^−/−^, *BRCA1*^−/−^*SHLD2*^−/−^, *BRCA1*^−/−^*SHLD3*^−/−^, and *BRCA1*^−/−^*REV7*^−/−^ RPE-1 cells. **(F)** RAD51 foci in EdU positive nuclei measured 3 hours after 5 Gy IR or 1 hour after 1 μM CTP treatment in WT, *BRCA1*^−/−^, *BRCA1*^−/−^*53BP1*^−/−^, *BRCA1*^−/−^*SHLD1*^−/−^, *BRCA1*^−/−^ *SHLD2*^−/−^, *BRCA1*^−/−^*SHLD3*^−/−^, and *BRCA1*^−/−^*REV7*^−/−^ RPE-1 cells (Mann-Whitney test, *P < 0.01, ****P < 0.0001; white dashed line represents the median, and black dashed lines represent the quartiles; ns, not significant).

Loss of BRCA1 leads to embryonic lethality, tumorigenesis, and hypersensitivity to agents that challenge the replication fork (46–48). In mice, co-deletion of 53BP1 and BRCA1 largely reverses these phenotypes (18, 19). Because 53BP1 limits the formation of recombinogenic ssDNA at canonical DSBs (49), it is presumed that hyper-resection unleashed in the absence of 53BP1 can restore HR in BRCA1-deficient cells (18, 19). To test whether this is true in the context of collapsed forks, we deleted 53BP1 in *BRCA1*^−/−^ cells expressing inducible nCas9^D^ (fig. S14A). Whereas nick-induced DSBs persisted for at least 16 hours in *BRCA1*^−/−^ cells, they were largely resolved by this time in *BRCA1*^−/−^*53BP1*^−/−^ cells (Fig. 5, A and B, and fig. S14, B and C). Hyper-resection was also decreased in *BRCA1*^−/−^*53BP1*^−/−^ cells (Fig. 5C) whereas RAD51 binding was restored (Fig. 5D). Thus, in contrast to canonical DSBs, 53BP1 does not block end resection in BRCA1-deficient cells but rather limits RAD51 filament formation at collapsed forks.

The Shieldin complex acts downstream of 53BP1 to counteract resection at canonical DSBs (14, 15). To test whether Shieldin also regulates RAD51 filament formation after resection, we compared the response of *BRCA1*^−/−^ RPE-1 cells lacking REV7, SHLD1, SHLD2, or SHLD3 with replication-coupled versus canonical DSBs. Like 53BP1 deficiency, loss of the Shieldin complex in *BRCA1*^−/−^ cells markedly increased RPA foci formation in response to IR but not CPT (Fig. 5E). However, compared with 53BP1 deficiency, loss of Shieldin only partially restored RAD51 filament formation in *BRCA1*^−/−^ cells treated with IR, and their impact was even milder in response to CPT (Fig. 5F). Thus, 53BP1 blocks the loading of RAD51 at collapsed forks in *BRCA1*^−/−^ cells, which is only modestly influenced by the downstream Shieldin complex.

The E3 ubiquitin ligase RNF168 acts redundantly with BRCA1 to promote RAD51 loading at canonical DSBs (12, 20, 50). BRCA1/53BP1-deficient mice lacking RNF168 are inviable, and derived cells exhibit PARPi hypersensitivity as well as a loss of IR-induced RAD51 foci formation (20). We found that loss of RNF168 also impaired RAD51 filament formation in *BRCA1*^−/−^*53BP1*^−/−^ cells treated with CPT (fig. S14D) or at site-specific nick-induced DSBs (fig. S14E). Thus, at both canonical and replication-coupled DSBs, RNF168 supports BRCA1-independent HR.

RNF168 ubiquitinates nucleosomal histone H2A on lysine 15 (H2AK15ub) at DSB sites (51, 52), a mark that is recognized by several DNA damage response factors including itself (53) and 53BP1 (54). This raises the possibility that 53BP1 may interfere with RNF168 recruitment to collapsed forks. RNF168 foci were barely detectable in WT and *BRCA1*^−/−^ cells treated with CPT (fig. S14F), consistent with recent proteomic analyses demonstrating that proteins involved in ubiquitin signaling are depleted at broken forks (55). However, RNF168 foci formation increased significantly in *BRCA1*^−/−^*53BP1*^−/−^ cells treated with CPT (fig. S14F). Thus, 53BP1 blocks BRCA1-independent RAD51 filament formation by limiting RNF168 accumulation at nick-induced DSBs.

## The dynamics of collapsed fork repair is conserved at diverse genomic loci

**Fig. 6.**
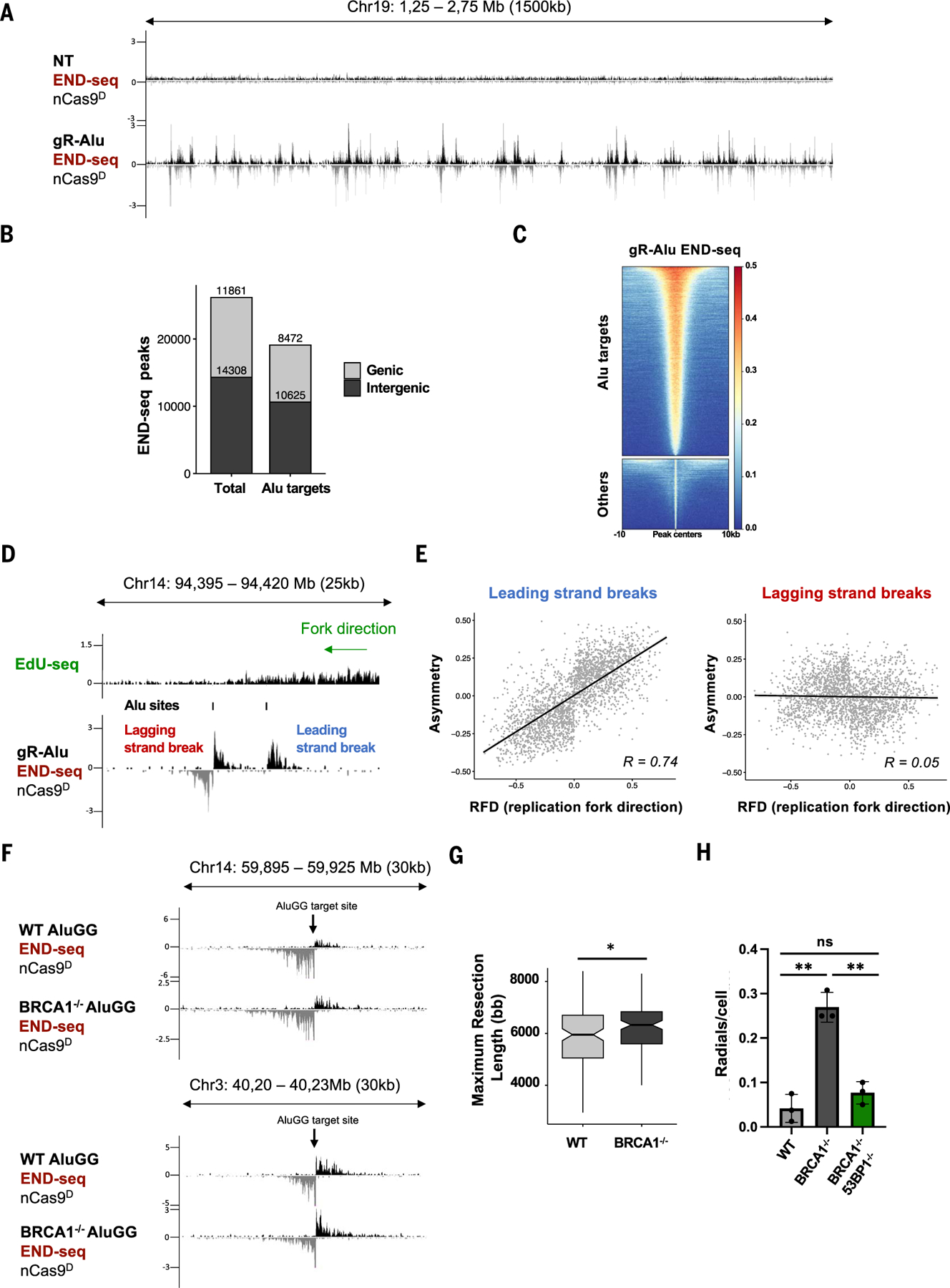
Generation of collapsed forks at diverse genomic loci. **(A)** Genome browser screenshots displaying END-seq signals in MCF10A cells that were either nontreated (NT) or expressing nCas9^D^/gR-Alu. **(B)** Genomic distribution of total and Alu-specific END-seq peaks in MCF10A cells expressing nCas9^D^/gR-Alu. **(C)** Heatmap of END-seq signals at Alu-specific and off-target (“Others”) sites. **(D)** Genome browser screenshot displaying EdU-seq and END-seq profiles. Both lagging- and leading-strand nick-induced DSBs are generated near a unidirectional fork. **(E)** Scatterplots showing the correlation between END-seq peak asymmetry and RFD of leading and lagging strand nCas9^D^/gR-Alu-induced breaks (Spearman correlation; correlation coefficient, *r*, of 0.74, for leading-strand breaks, and 0.05, for lagging-strand breaks). **(F)** Genome browser screenshot displaying END-seq signals at nick-induced DSBs generated by nCas9^D^/AluGG in WT and *BRCA1*^−/−^ RPE-1 cells. **(G)** Box plot showing maximum resection lengths in WT and *BRCA1*^−/−^ cells at AluGG-induced DSBs (Wilcoxon test, *P = 0.033). **(H)** Radial chromosomes in metaphase spreads from WT, *BRCA1*^−/−^, and *BRCA1*^−/−^*53BP1*^−/−^ cells expressing nCas9^D^/AluGG (*n* =3 independent replicates, >40 metaphases analyzed per replicate, **P < 0.005 unpaired *t* test). Radials in Dox-induced cells were determined by subtracting the radials present before nCas9^D^/AluGG induction.

Recently, degenerate guide RNAs have been designed that target Cas9 to thousands of epigenetically diverse genomic sites (56, 57). For example, a single sgRNA was used to edit Alu elements (gR-Alu), which are present at >100,000 locations in the human genome (56). Because the length of individual Alu element (~280 bp) is much shorter than the average resection tract of a collapsed fork, we hypothesized that END-seq could be used to map nick-induced DSBs at these sites, allowing simultaneous tracking of end processing and repair at multiple loci. Indeed, after inducing the expression of nCas9^D^/gR-Alu in MCF10A cells (56), we detected 26,169 END-seq peaks, 73% of which aligned with annotated Alu elements (Fig. 6, A to C). DSBs were located throughout the genome, exhibiting relatively balanced representation between genic and intergenic regions (Fig. 6, B and C). Moreover, seDSB and deDSBs were clearly distinguishable (Fig. 6D). For a given nick site, we derived replication fork directionality (RFD) from OK-seq and used it to predict whether it would generate a leading- or lagging-strand collision event. For example, if a nick was targeted to the Crick strand in which the RFD value was >0 (<0), it would be designated as a lagging-strand (leading-strand) nick. We found that DSB asymmetry at predicted leading-strand collapse sites was directly proportional to the RFD value (i.e., greater asymmetry at regions with more unidirectional forks) (Fig. 6E). By contrast, DSB asymmetry at lagging-strand nick sites did not correlate with RFD (Fig. 6E). These data demonstrate that strand asymmetrical generation of nick-induced DSBs is a general phenomenon that is largely agnostic to (epi)genomic context.

PARP inhibitors cause an increase in single-strand breaks (SSBs) in BRCA1/2 defective cells, which are converted to DSBs after collision with the replication fork (58, 59). Replication-coupled DSBs are then processed by NHEJ into lethal repair products including radial chromosomes that are readily identifiable in metaphase spreads (14). However, we were unable to collect metaphase spreads from *BRCA1*^−/−^ cells expressing nCas9^D^/gR-Alu, likely owing to the overwhelming number of unrepaired DSBs. We therefore deployed a different degenerate sgRNA that targets fewer (~126) Alu elements (AluGG) (57). We confirmed that BRCA1 is dispensable for DSB end resection at AluGG target sites (Fig. 6F). Indeed, the median resection length was greater in *BRCA1*^−/−^ than in WT [5950 bp (WT) versus 6325 bp (*BRCA1*^−/−^); Fig. 6G]. Both WT and *BRCA1*^−/−^ cells could reach mitosis under these conditions. We found that the frequency of radial fusions induced by nCas9^D^/AluGG was elevated sixfold in *BRCA1*^−/−^ compared with WT, and it was largely rescued by loss of *53BP1* (Fig. 6H). Overall, these data are consistent with our analysis of individually broken replication forks and reinforce the notion that DNA nicks are lesions with direct relevance to genome instability, particularly in cells lacking functional HR.

## Discussion

The ability to synchronously induce site-specific nicks has made it possible to systematically model the consequences of their collision with replication forks. Our analysis of the structure, fate, and recovery of collapsed replication forks has revealed an unexpected asymmetry between leading- and lagging-strand fork collapse and uncovered differential processing of nick-induced DSBs versus canonical DSBs. As collapsed forks are thought to be responsible for most of the endogenous DSBs (60), are targeted during chemo-therapy (8), and are generated as genotoxic by-products of nCas9-based prime- and base-editing technologies (61, 62), our system to detect, quantify, and track nick-induced DSBs should serve as a blueprint for a deeper understanding of HR and its genome engineering applications.

### Structural determinants of nick-induced DSBs

The distinct repair outcomes and end structures associated with leading-versus lagging-strand fork collapse are strongly influenced by the nature of the single-strand interruption and whether they are protein-bound or concealed from genome surveillance factors. Any nick that is not occluded is likely to be rapidly detected and repaired by the SSB repair machinery before the arrival of the replication fork, thereby avoiding catastrophic DSBs (60). A good example of this is when nCas9^H^ cleaves the strand that is not engaged with the Cas9/sgRNA complex (fig. S1C), the resulting exposed 3′ end of the nick (27) is recognized by the HIRAN domain of HLTF whose translocase activity evicts nCas9^H^ from DNA. The subsequent accessibility of the nick to the SSB repair machinery likely reduces the frequency of collapsed forks. Conversely, the nick generated by nCas9^D^ is tightly engaged within the Cas9/sgRNA complex and therefore is obscured from the cellular surveillance and SSB repair pathways. Upon leading-strand fork collapse, CMG, which encircles the leading-strand template, “runs off” the end of the nCas9-bound nick (6), resulting in a highly resected seDSB.

When colliding with a nCas9^D^-induced lagging-strand nick, the replisome traverses the “concealed” nick without CMG dissociation, generating a deDSB. The availability of the “second” end may explain why HR is more strongly stimulated when a nick is generated on the lagging-strand template compared with the leading-strand template (17). Because protein-bound nicks can arise under multiple conditions, including stabilized topoisomerase-DNA cleavage complexes (63), blocked RNA polymerases forming R-loops (64), trapped PARP-DNA complexes (60), and during AAV viral integration (65), it is possible that collisions with certain endogenous adducts will also yield deDSBs. Notably, using the Flp-nick system, which mimics a covalent topoisomerase 1–DNA cleavage complex, Elango and colleagues recently demonstrated that Flp-induced nicks generate deDSB intermediates that are resolved by HR in a manner independent of BRCA1’s function in end resection (66), consistent with our findings.

### Role of the converging fork in genome integrity

The importance of the converging fork in the generation of DSB structures at DNA nick sites remains unclear. Traversal of a lagging-strand nCas9^D^ nick by the replisome creates a deDSB upon nCas9^D^ dissociation, obviating the need for a converging fork. In contrast, fork convergence converts seDSBs to deDSBs upon leading-strand collapse in RAD51-, BRCA1-, and BRCA2-deficient cells. These resected deDSBs likely contribute to the genomic scars, including microhomology-mediated indels, that are characteristic of HR-deficient tumors (67–71). Additionally, we note that the formation of deDSBs at leading-strand collapse in HR-deficient cells implies that the gap created on the lagging strand where the last Okazaki fragment is generated remains unsealed before the arrival of the converging fork, thereby avoiding re-replication (6, 39). Although the mechanism that prevents gap repair in this setting remains unclear, it is possible that it could be related to BRCA1 and BRCA2’s proposed role in gap suppression (72, 73).

It has also been suggested that the converging fork could influence the repair of collapsed forks, for instance by terminating the migrating D-loop of an invading seDSB (5, 74). Accordingly, we observed that the extent of RPA binding beyond the nick site, indicative of ssDNA generated during strand invasion (38), is limited by the converging fork. The converging fork may thus favor short-tract DNA recombination that is more likely to be error free (5).

### The role of BRCA1 in the repair of nick-induced DSBs

Nick-induced repair junctions in BRCA1-deficient cells exhibit longer deletions and increased microhomology usage than in WT cells, whereas the opposite was observed at canonical DSBs (22). These distinct repair outcomes can be explained by our finding that whereas BRCA1 facilitates end resection at deDSBs induced outside of the context of replication, resection at replication-coupled DSBs is BRCA1 independent.

A recent study reported that 53BP1/Shieldin-mediated fill-in synthesis, while essential for the joining of AID-dependent canonical DSBs during class-switch recombination, is largely dispensable for HR suppression in BRCA1-deficient cells (75). This is consistent with our finding that 53BP1 suppresses BRCA1-independent RAD51 loading at replication-coupled DSBs independently of Shieldin and without affecting resection. Efficient restoration of HR in *BRCA1*^−/−^*53BP1*^−/−^ cells requires RNF168 (12, 20). Given that RNF168 interacts with PALB2 (50) and both RNF168 and 53BP1 associate with H2AK15ub (53, 54, 76, 77), we envision that 53BP1 inhibits PALB2/RAD51 recruitment through competition with RNF168. Consistent with this idea, we found that RAD51 filament formation in *BRCA1*^−/−^*53BP1*^−/−^ cells correlates with the accumulation of RNF168 at DSBs. Moreover, because 53BP1 interacts with H2AK15ub independently of Shieldin, this model would help explain why HR suppression in BRCA1-deficient cells is strongly reliant on 53BP1 but less so on Shieldin (75).

### End resection at collapsed replication forks versus canonical DSBs

What distinguishes broken replication forks from canonical DSBs such that they are processed in a distinct manner? One possibility could be differential accessibility to cellular nucleases that process damaged chromatin in preparation for HR. Because the resection machinery is recruited to canonical DSBs de novo, along with other DNA damage response proteins including BRCA1 and 53BP1/Shieldin, the pro- and anti-resection activities of BRCA1 and 53BP1/Shieldin may directly compete to regulate nucleolytic processing and DSB repair choice. Conversely, proteomic analyses suggest that resection factors such as CtIP, MRE11, EXO1, and DNA2 travel with unperturbed replication forks (78). They are therefore poised to respond immediately to fork collapse and breakage. By having instantaneous access to broken forks, these nucleases commit cells to HR, which in turn minimizes NHEJ and other mutagenic pathways. Indeed, whereas loss of NHEJ elevates HR at canonical DSBs (79), NHEJ does not compete with HR induced by nCas9- or Flp-induced nicks (17, 66). Our finding that collapsed forks are resected as soon as DSBs arise is consistent with the idea that their repair is tightly controlled to favor HR. In the absence of subsequent BRCA1/BRCA2-mediated RAD51 loading and strand invasion, resection becomes hyperactivated, suggesting that RAD51 nucleofilament formation imparts a negative feedback on end resection. A similar phenomenon is observed at programmed DNA breaks during meiosis, wherein spermatocytes lacking the DMC1 recombinase or those deficient in RAD51/DMC1 foci formation owing to a BRCA1 mutation exhibit hyper-resection (80, 81). This likely reflects the fact that repair of both broken replication forks and meiotic DSBs is tightly coupled to HR.

## Materials and methods

### Cell culture and treatments

MCF10A cells were cultured in a 1:1 mixture of Dulbecco’s modified Eagle’s medium (DMEM) and Ham’s F12 medium (Thermo Fisher Scientific), supplemented with 5% horse serum (Thermo Fisher Scientific), 100 ng/ml cholera toxin (Sigma-Aldrich), 0.5 μg/ml hydrocortisone (Sigma-Aldrich), 10 μg/ml insulin (Sigma-Aldrich), 20 ng/ml human epidermal growth factor (Sigma-Aldrich), and Pen-Strep (Gibco). RPE-1 WT or *BRCA1*^−/−^ cells generated using all-in-one plasmids containing CasD10A and two sgRNAs targeting exon9 of BRCA1 [a gift from S. P. Jackson (21)] were cultured in a 1:1 mixture of DMEM and Ham’s F12 medium (Thermo Fisher Scientific), supplemented with 10% tetracycline-negative FBS bovine serum (R&D Systems), and penicillin-streptomycin (Gibco). To produce MCF10A nCas9^D^, nCas9^H^, and dCas9 and RPE-1 WT and *BRCA1*^−/−^ nCas9^D^ and Cas9 doxycycline-inducible cell lines, we generated lentivirus in human embryonic kidney 293T cells. The supernatant containing the virus particles was filtered, and MCF10A or RPE-1 cells were infected and selected with 10 μg/ml blasticidin (ThermoFisher). To confirm nCas9^D^, nCas9^H^, dCas9, and Cas9 expression, constructs were induced by treating cells with 3 μg/ml doxycycline followed by Western blot detection. Guide RNAs (table S1) were cloned in the Lenti-Guide-NLS–GFP (82, 83), and MCF10A or RPE-1 nCas9D, nCas9H, dCas9, or Cas9 cells containing the guide constructs were selected using 3 μg/ml puromycin. To arrest cells in G_1_ and induce Cas9, palbociclib (Selleckchem, #S1116; 1 μM for MCF10A and 0.5 μM for RPE-1) was added together with 3 μg/ml doxycycline (Dox) for 20 hours. To synchronously release the culture into S phase, cells were washed three times with 1X DPBS (Corning), released in fresh media, and collected at the indicated time points. In the experiments where collapsed fork resolution was analyzed, palbociclib was re-added 6 hours after release to avoid new cells entering into S phase. For S1-END-seq and END-seq experiments in G_1_-arrested cells, 5 μM palbociclib was used for 24 hours for G_1_ arrest. RPE-1 WT, *BRCA1*^−/−^, *BRCA1*^−/−^*53BP1*^−/−^, *BRCA1*^−/−^*SHLD1*^−/−^, *BRCA1*^−/−^*SHLD2*^−/−^, *BRCA1*^−/−^ *SHLD3*^−/−^, and *BRCA1*^−/−^*REV7*^−/−^ cells (a gift from D. Durocher) were cultured as described previously (82). MDA-MB-436 (BRCA1mut) and MDA-MB-436 + BRCA1WT (a gift from N. Johnson), were cultured as described previously (45).

### Inducible Cas9 constructs—nCas^D^, nCas9^H^, dCas9, WT Cas9, and Ct-degron nCas9^D^

The inducible nCas9^D^ system was generated as described previously (83). Inducible nCas9^H^ and dCas9 were made by isothermal assembly using fragments with PCR-induced mutagenesis. Briefly, nCas9^H^ was made by digesting Addgene-83481 with *Nhe*I/*BamH*I and then assembling it with two PCR fragments, with the second fragment carrying the H840 mutation. In the case of dCas9, the construct was generated by digesting Addgene-83481 with NheI/BamHI and then assembling it with a PCR fragment from D10A nickase and a second fragment carrying the H840A mutation. Inducible canonical Cas9 was obtained from Addgene (83481). C-terminal degron nCas9^D^ (nCas9 D10A Ct degron) was generated by first digesting nCas9^D^ with BamHI, amplifying the dTAG degron from the dTAG-2A-Puro plasmid (Addgene-91796) using the following primers: Forward GCCAGGCAAAAAAGAAAAAGGGT-GGCGGTGGCTCGGGCGG, and Reverse AAAA-GGCGCAACCCCAACCCCGGATCCTTAGCCAGA-GCCTTCCAGTTTTA, and cloning them together using isothermal assembly.

### Flow cytometry

To measure DNA synthesis, MCF10A or RPE-1 cells were pulsed with 10 μM of EdU for 30 min at 37°C and stained using the Click-IT EdU Alexa Fluor 488 Flow Cytometry Assay Kit according to the manufacturer’s instructions (ThermoFisher). DNA content was measured with 4′,6-diamidino-2-phenylindole (DAPI) 1 μg/ml. Data were acquired on CytoFLEX (Beckman Coulter) and analyzed using CytExpert software.

### TrAEL-seq

TrAEL-seq was adapted from the published protocol (26). Briefly, five million to six million cells were harvested in phosphate-buffered saline (PBS) and embedded in two agarose plugs before proteinase K and RNaseA treatment. Genomic DNA ends were A-tailed using terminal deoxynucleotidyl transferase (NEB M0315L), 5′ adenylated with TrAEL-seq adaptor 1, and ligated using T4 RNA ligase 2 truncated KQ (NEB M0373L). The agarose plugs were then melted using β-agarase (NEB M0392L), and the DNA fragmented by sonication. Second-end adaptor ligation and library preparation were performed using the same protocol as END-seq. The samples were sequenced with the NextSeq2000 sequencer.

### END-seq, S1-END-seq, and EdU-seq

Protocols for END-seq and S1-END-seq have been described previously (83–85). Briefly, 15 million MCF10A or RPE-1 cells were embedded in agarose plugs and treated with proteinase K for1 hour at 50°C and then for7 hours at 37°C. Plugs were washed in washing buffer (10 mM Tris-HCl, pH 8.0, 50 mM EDTA) and then in TE (10 mM Tris-HCl, pH 8.0, 1 mM EDTA). RNase A treatment was performed for 1 hour at 37°C. DNA DSBs were blunted with exonuclease VII (NEB) for 1 hour at 37°C and exonuclease T (NEB) for 45 min at 24°C. Adapter ligation, DNA sonication, and library preparation were performed as described previously (24, 28). For S1-END-seq experiments, plugs were washed once with washing buffer, then twice with elution buffer (10 mM Tris, pH 8.0), and equilibrated with two washes of S1 nuclease buffer (40 mM sodium acetate pH 4.5, 300 mM NaCl, 2 mM ZnSO_4_). After the washes, plugs were incubated at 37 °C for 30 min with 200 U of S1 nuclease (Thermo Fisher) in 100 μl 1X S1 nuclease buffer per plug. DNA end blunting, adapter ligation, DNA sonication, and library preparation followed the same protocol as END-seq. For EdU-seq, MCF10A cells were arrested in G_1_ using 1 μM palbociclib (Selleckchem, #S1116) for 20 hours and released for 4 hours in the presence of 4 μM APH (Sigma). Cells were pelleted and fixed in 90% methanol overnight at −20°C. Cells were washed and permeabilized on ice with 0.2% Triton X-100 in PBS for 10 min and then washed with 1X DPBS (Corning). For the Click-IT reaction, cell pellets were resuspended in PBS, 10 μM biotin azide (ThermoFisher Cat# B10184), 200 μM CuSO_4_ (Sigma), and 10 mM sodium ascorbate (Sigma) for 2 hours in the dark at room temperature. DNA sonication and library preparation were performed as described previously (24). Sequencing was performed on the Illumina NextSeq 2000 (100 bp single-end reads) or NextSeq 550 (75 bp single-end reads).

### RPA and RAD51 ssDNA ChIP-Seq

Twenty million cells were harvested and fixed for 10 min in 1% formaldehyde. Cells were lysed with ice-cold RIPA buffer (10 mM Tris HCl pH 7.5, 1 mM EDTA, 0.1% SDS, 0.1% sodium deoxycholate, 1% Triton X-100) supplemented with EDTA-free protease cocktail inhibitor. Chromatin was sheared using the Covaris S220 sonicator at duty cycle 20%, peak incident power 175, cycle/burst 200 for 45 min. Sheared chromatin was cleared by centrifugation, and ChIP was performed as described previously (37, 86). Briefly, the lysate was precleared, and DNA was captured by incubating lysates with 10 μg of Anti-RPA32/RPA2 antibody (Abcam, ab10359) or RAD51 antibody (Abcam, ab176458) bound to Dynabeads protein A (Invitrogen) overnight at 4°C. Beads were separated using a magnetic separator (DynaMag-2 Invitrogen) and washed twice with cold RIPA buffer, twice with RIPA buffer containing 0.3 M NaCl, twice with LiCl buffer (0.25 M LiCl, 0.5% Igepal-630, 0.5% sodium deoxycholate), once with TE (10 mM Tris pH 8.0, 1 mM EDTA) plus 0.2% Triton X-100, and once with TE, and resuspended in TE with 1% SDS and proteinase K. DNA was eluted by decrosslinking at 65°C in thermomixer for 4 hours. Purified DNA was quantified, and 100 ng ssDNA was used for Illumina sequencing. For ssDNA kinetic enrichment, the DNA was denatured at 95°C for 3 min and cooled down to room temperature before adapter ligation. Library concentration was determined with KAPA Library Quantification Kit for Illumina Platforms (Kapa Biosystems). Sequencing was performed on the Illumina NextSeq 2000 (100 bp single-end reads).

### Amplicon-seq

To perform amplicon-seq, the genomic DNA from RPE-1 cells was extracted after 3 days of Dox treatment and amplified in a two-round PCR reaction. The gDNA was initially amplified using the primers: Forward 5′-TCGTCGGCA-GCGTCAGATGTGTATAAGAGACAG-[locus-specific sequence] and Reverse 5′-GTCTCGTGG-GCTCGGAGATGTGTATAAGAGACAG-[locus-specific sequence] (table S2). Reactions were performed using KAPA HiFi HotStart ReadyMix (Roche) using the following conditions: 3 min at 95°C, 25 cycles of 30 s at 95°C, 15 s at 60°C, 30 s at 72°C, followed by a single 5-min step at 72°C. For the second PCR reaction, 50 ng of purified DNA product was used as a template and amplified using Nextera XT Index Primer 1 (N7XX) and Nextera XT Index Primer 2 (N5XX) (Illumina). Reactions were again performed using KAPA HiFi HotStart ReadyMix (Roche) under the following conditions: 3 min at 95°C, eight cycles of 30s at 95°C, 30s at 55°C, 30s at 72°C, followed by a single 5-min step at 72°C. The resulting libraries were purified, and library concentration was determined with KAPA Library Quantification Kit for Illumina Platforms (Kapa Biosystems). Sequencing was performed on the Illumina NextSeq 550 (150 bp paired-end reads). Paired-end reads were first merged together by fastq-join (version: 1.3.1) (87). Then the merged reads were mapped to the human genome (hg19) using bwa-mem2 (version 2.2.1) (88). Deletions and microhomology inthe mapped reads were called by a custom Perl script.

### Single-molecule fork collapse assay

The single-molecule reactions were carried out as previously described (6, 89). Guide RNA for single-molecule experiments was prepared by annealing AltR CRISPR-Cas9 tracrRNA, ATTO 550 (IDT) with 10-fold excess Alt-R CRISPR-Cas9-crRNA (IDT) in 1X Annealing Buffer (IDT) to yield 4 μM guide RNA. The guide RNAs were designed to target two sites on the same strand of the 30-kb single-molecule DNA. The Cas9 ribonucleoprotein (RNP) was formed by incubating 25 pmol Alt-R s.p. Cas9 D10A Nickase (IDT) with 2 pmol guide RNA in Cas9 binding buffer (20 mM Tris, pH 7.5, 100 mM KCl, 5 mM MgCl_2_, 1 mM DTT, 5% glycerol) in the dark for 20 min before use.

Flow cells were assembled with coverslips passivated with 10% biotin-PEG-SVA and m-PEG-SVA MW5000 (Layson Bio). All buffers were degassed for 1 hour before use. Flow cells were incubated with 0.2 mg/ml streptavidin (Sigma) for 15 min. Flow cells were then washed with 500 μl DNA Blocking Buffer [20 mM Tris, pH 7.5, 50 mM NaCl, 2 mM EDTA, 0.2 mg/ml bovine serum albumin (BSA)] + 0.5% Tween20 at 500 μl/min. DNA was then double tethered to the coverslip by flowing in 500 μl of DNA solution containing 67 pg/μl DNA that was biotinylated at each end at 100 μl/min. Next, the flow cell was washed with 60 μl Cas9 binding buffer (20 mM Tris, pH 7.5, 100 mM KCl, 5 mM MgCl_2_,1 mM DTT, 5% glycerol) at 20 μl/min. Cas9 RNP (2 nM, described above) solution was then added at 20 μl/min. Nonspecifically bound nCas9 was removed from the tethered DNA by washing with a solution containing 0.5 M NaCl (20 mM Tris, pH 7.5, 500 mM NaCl, 5 mM MgCl_2_, 1 mM DTT, 5% glycerol) at 20 μl/min. The flow cells were then equilibrated with 60 μl Cas9 buffer at 20 μl/min, before adding 30 μl of 200 nM Sytox Green (Thermo Fisher Cat # S7020) in Cas9 binding buffer at 20 μl/min to image the DNA and nCas9. Each field of view (FOV) was imaged for both Sytox and nCas9Atto550 using alternating 488 nm and 561 nm laser excitation. The Sytox was washed off the DNA, and the flow cell was equilibrated with 150 μl 1X ELB-sucrose (10 mM HEPES-KOH, pH 7.7, 2.5 mM MgCl_2_, 50 mM KCl, 250 mM sucrose) at 10 μl/min.

*Xenopus* egg extracts were immunodepleted of GINS as previously described (6, 89). The Cas9 RNP-bound DNA was then licensed with 20 μl high-speed supernatant (HSS) extract for 6 min. Replication was then initiated by flowing in 20 μl GINS-depleted HSS/NPE mix that included 0.01 mg/ml recombinant GINS^AF647^,2 μM Fen1-mKikGR D179A, and 3.7 nM nCas9^Atto550^ at 10 μl/min. After 4 min, excess GINS^AF647^ and nCas9 were removed from the flow cell by flowing in additional 50 μl GINS-depleted HSS/NPE mix that included 2 μM Fen1-mKikGR D179A at 10 μl/min. Images of Fen1-mKikGR, nCas9Atto550, and GINSAF647 were acquired every minute for 1 hour by cycling among the 488-nm [64°–65° total internal reflection fluorescence (TIRF) angle, 0.23 mW power, 100 ms exposure, 999 electron multiplying gain], 561-nm (61°–66° TIRF angle, 0.35 mW power, 100 ms exposure, 999 EM GAIN), and 647-nm (61°–63° TIRF angle, 0.15 mW power, 100 ms exposure, 999 electron multiplying gain) lasers at each of the fields of view. Specific microscope configurations were previously described (89). Movies were collected using NIS Elements software and saved as nd2 files. All image analysis was performed using a combination of freely available and custom MATLAB scripts, as previously described (6).

### Immunofluorescence

Cells were seeded on 12-mm round glass coverslips and allowed to attach overnight. Cells were then incubated with 10 μM EdU for 30 min. Cells were then treated with dimethyl sulfoxide or 1 μM CPT for 1 hour. Alternatively, cells were subjected to γ-irradiation [5 gray (Gy)] using a cesium-137 source (Mark 1 irradiator, JL Shepherd) and allowed to recover for 3 hours. Before fixation in 4% paraformaldehyde (10 min), samples were first pre-extracted (20 mM HEPES, 50 mM NaCl, 3 mM MgCl_2_, 0.3 M sucrose, 0.2% Triton X-100) on ice for 5 min to remove soluble nuclear proteins. Fixed samples were then permeabilized (0.5% Triton X-100, 5 min), blocked (2% BSA/PBS, 30 min), and incubated with primary antibodies recognizing RPA2 (1:1000, Cell Signaling #2208) and RAD51 (1:250, Millipore, ABE257) in blocking solution (2 hours, 37°C). Detection was accomplished with appropriate fluorochrome-conjugated secondary antibodies (Invitrogen). Next, click-IT chemistry was performed following the manufacturer’s instructions (Thermo Fisher Scientific), and DNA was counterstained with DAPI. Fluorescence images were captured at 40× magnification on a Lionheart LX automated microscope (BioTek Instruments). Quantification of nuclear foci was performed using the Gen5 spot analysis software (BioTek Instruments).

### Western blotting

Cells were pelleted, washed with 1x DPBS, and lysed in 50 mM Tris-HCl (pH 7.5), 200 mM NaCl, 5% Tween, 0.5% NP-40, 2 mM phenylmethylsulfonyl fluoride, 2 mM β-glycerophosphate disodium salt hydrate (Sigma), and one tablet of cOmplete mini protease inhibitor cocktail (Roche 11836153001). Fifty micrograms of protein lysates were loaded into SDS–polyacrylamide gel electrophoresis mini-gels (Bio-Rad). After protein transfer, the nitrocellulose membranes (BioRad) were incubated with anti-Cas9 antibody (Novus, NBP280679), anti-HLTF (Abcam, ab17984), anti-POLD3 (Abnova, H00010714-M01), anti-RAD52 (Santa Cruz, sc-365341), anti-RAD51 (Abcam, ab176458), anti-BRCA1 (Millipore, OP92), anti-CtIP (Active motif, 61942), anti-53BP1 antibody (Novus, #NB100-305), anti-Tubulin (Sigma, #T-5168), anti-GAPDH (Cell Signaling, 2118S) overnight at 4°C. Fluorescent secondary antibody anti-mouse or anti-rabbit IRDye 800CW or 680RD (LI-COR Biosciences) was incubated for 1 hour at room temperature. Image acquisition was performed using an Odyssey CLx machine (LI-COR Biosciences)

### Metaphase spreads

RPE-1 WT, *BRCA1*^−/−^, and *BRCA1*^−/−^/*53BP1*^−/−^ cells were infected using lentivirus particles with Lenti-Guide-NLS–GFP containing sgA-luGG sequence. After 24 hours, the virus supernatant was removed, and fresh media containing 3 μg/ml doxycycline was added to induce nCas9^D^. Colcemid (0.04 μg/ml, Roche) was added 24 hours after Dox induction, and cells were collected 16 hours later. Metaphase chromosome spreads were prepared as previously described (20). Images were captured at 63× magnification using the Metafer automated scanning and imaging platform (MetaSystems). More than forty metaphases were scored for the presence of radial aberrations.

### Quantitative PCR

To measure nick efficiency by qPCR, cells were treated with 5 μM palbociclib (G_1_ arrest) and 3 μg/ml Dox (nickase induction) for 24 hours, and genomic DNA was extracted. For qPCR reactions, 5 μg of gDNA from each construct was initially subjected to S1 nuclease digestion (Thermo Scientific) using 200 U of the enzyme in 100 μl S1 buffer for 30 min at 37°C. Fifty nanograms of the treated genomic DNA was used for analysis on the CFX96 Real-Time PCR Detection System (Bio-Rad, Hercules, CA, USA) using iTaq Universal SYBR Green Supermix (Bio-Rad). The PCR primers are listed in table S3. *ACTB* served as an internal control for all samples.

### siRNA-mediated knockdowns

The following siRNAs from Horizon Discovery were used: ON-TARGETplus Human siRNA targeting: HLTF (L-006448-00-0005), RAD51 (J-003530-12), POLD3 (L-026692-01, SMARTpool), RAD52 (J-011760-06, SMARTpool), BRCA2 (L-003462-00, SMARTpool), CtIP (90) (custom siRNA, 5′ GCUAAAACAGGAACGAAUCUU 3′, Horizon Discovery), RNF168 (M-007152-03-0020, SMARTpool), as well as nontargeting control pool (D-001810-01). Cells were transfected using DharmaFECT Transfection Reagent (Horizon Discovery) according to the manufacturer’s instructions. Depletion of proteins was confirmed by Western blotting.

### Generation of 53BP1 KO cells

53BP1 knockout in RPE-1 *BRCA1*^−/−^ cells was generated by RNP-based gene editing technology using synthetic guide RNAs and Cas9 protein (Synthego). Briefly, sgRNAs were hydrated in TE, and RNP complex was assembled by incubating 300 pmol of sgRNA and 40 pmol of Cas9 protein in 10 μl at room temperature for 10 min. RNP complex was added to 90 μl of cell suspension containing 0.5 × 10^6^ cells and electroporated at 125 V for 5 ms (NEPA21, NEPAGENE). Electroporated cells were plated on a six-well plate and single-cloned. Synthetic modified RNA for TP53BP1: sgRNA1,AGTGCTCAGATTCCCAGTCA; sgRNA2, GATCGGAAAGCATCAGGAGA.

### Genome alignment

For the END-seq, raw reads from Illumina NextSeq 2000/5500 were aligned to the human genome (hg19) or mouse (mm10) using Bowtie (v1.3.1) (91) with -n 3 -l 50 -k 1. For ChIP-seq, Illumina adapter and low-quality reads were trimmed using Trimmomatic (v0.39) (92). Processed reads were aligned to the human genome (hg19) using Bowtie2 (v2.2.5.1) (93) with the default parameter. Samtools (v1.5.1) (94) functions “view” and “sort” were used to convert and sort the aligned sam files to sorted bam files. Reads from the mitochondria and blacklisted regions were removed for further downstream analysis. Bam files were converted to bed files using the bedtools (v2.3.0) (95) bamTobed command.

### END-seq spike-in normalization

For comparison between samples, a spike-in control (mouse genome) was added to END-seq samples that consists of a G_1_-arrested Ableson transformed pre-B cell line (Lig4^−/−)^ carrying a single zinc finger–induced DSB at the TCRβ enhancer. This site is expected to break in all spike-in cells, which were mixed in at a 20% frequency with MCF10A or RPE-1 cells. For spike-in normalization, reads from the samples were aligned to both human and mouse genomes. The function “genomecov” from the bedtools was used to normalized read density (reads per million, RPM). The scaling factor for the spike-in normalization was calculated using the number of reads mapped to the spike-in locus of the mouse genome and the total number of reads mapped to the human genome. To generate the spike-in normalized reads, the normalized read density (RPM) was divided by the scaling factor.

### Calculation of RFD values

The processing pipeline for TrAEL-seq was adapted from the publication (26). UMI barcodes were first extracted by UMItools (96), and sequencing reads without ligation of adenosine nucleotides were discarded. For the remaining reads, up to 3 Ts at the start of the reads were removed. Sequencing adapters were then trimmed using Trimmomatic (92). Low-quality G-tracts at the 3′ ends of reads were removed by cutadapt (97). The processed reads were aligned to the human genome (hg19) using Bowtie2 (93). The bam files were fed into the OKseqHMM R package (https://github.com/CL-CHEN-Lab/OK-Seq) (25) to generate RFD values for each TrAEL-seq sample.

### Analysis of END-seq peak asymmetry

END-seq peaks were called using macs2 (parameters: –broad –nolambda –nomodel –shift −50 –extsize 100) (98). The peaks were further filtered by signal values > 3 and *q* values < 0.001. Because there are ~100,000 sgAlu target sites (maximum 1 mismatch) in the human genome (~1 per 32 kb), several sites are closely spaced. To remove the influence of multi-nicking events, we required that each END-seq peak contain only one target site. In addition, each peak should be at least 8 kb away from other target sites. Finally, 5581 peaks were qualified for peak asymmetry analysis. For each qualified peak we counted the numbers of reads falling with 3 kb to the left and right side of each nick, respectively. The peak asymmetry value was then calculated using the following formula

$$\text{Peak}\;\text{asymmentry}=0.5-{\text{Reads}}_{\text{right}}/\left({\text{Reads}}_{\text{left}}+{\text{Reads}}_{\text{right}}\right)$$

The graphs of correlation between peak asymmetry values and RFD values were generated using the ggscatter function from the ggpubr R package [Kassambara A (2023). *ggpubr: 'ggplot2' Based Publication Ready Plots*. R package version 0.6.0, https://rpkgs.datanovia.com/ggpubr/].

### Calculation of resection length

For the unique sgRNA (sg1/sg2/sg3/sg4/sg5/sg6), the “findpeaks” function from the R package pracma was used to identify the peak boundaries. The threshold for the background signal was determined by visually inspecting the peak in all locations for each sample. Once the boundaries were detected, the distance from the nick position and peak ends was measured to determine the resection length. For END-seq peak intensity quantification, the total signal (number of reads) ± 30 kb around the nick sites was measured.

For the degenerate sgRNA (AluGG), to quantify the resection length, a sliding window containing twelve 50-bp bins starting from each nick was used. The average END-seq signal for 50-bp bins within 7 to 9 kb from the nick was used as background. Starting from the nick, if more than eight bins within one sliding window had signals lower than the background, the last bin within this window (which had a signal higher than the background) was used to define the maximum resection endpoint.

### Visualization

Aligned-reads bed files were first converted to BedGraph files using bedtools genomecov. The BedGraph files were then converted to BigWig files using BedGraphToBigWig. Genome browser profiles were normalized to the library size (RPM). Visualization of genomic profiles was done by UCSC genome browser (99). In the experiments where repair is analyzed within the same genotype or between different genotypes, the spike-in normalized reads were used for visualization in the genome browser. The R package and GraphPadPrism were used to generate the bar plots.

## Supplementary Material

MDAR

supp

## Data Availability

Primary data used in this manuscript have been deposited to the Sequence Read Archive under BioProject ID PRJNA1121638. All other data are available in the main text or the supplementary materials.
