# Supplementary material for: Structure and repair of replication-coupled DNA breaks
Source: Science. Author manuscript; Available in PMC 2024 Dec 5. (PMC11620331; doi:10.1126/science.ado3867)
Supplement: supp [file NIHMS2033690-supplement-supp.pdf]

Science AAAS

Supplementary Materials for

Structure and repair of replication-coupled DNA breaks

Raphael Pavani *et al*.

Corresponding author: André Nussenzweig, andre_nussenzweig@nih.gov

Science **385**, eado3867 (2024)
DOI: 10.1126/science.ado3867

The PDF file includes:

Figs. S1 to S14
Tables S1 to S3

Other Supplementary Material for this manuscript includes the following:

MDAR Reproducibility Checklist

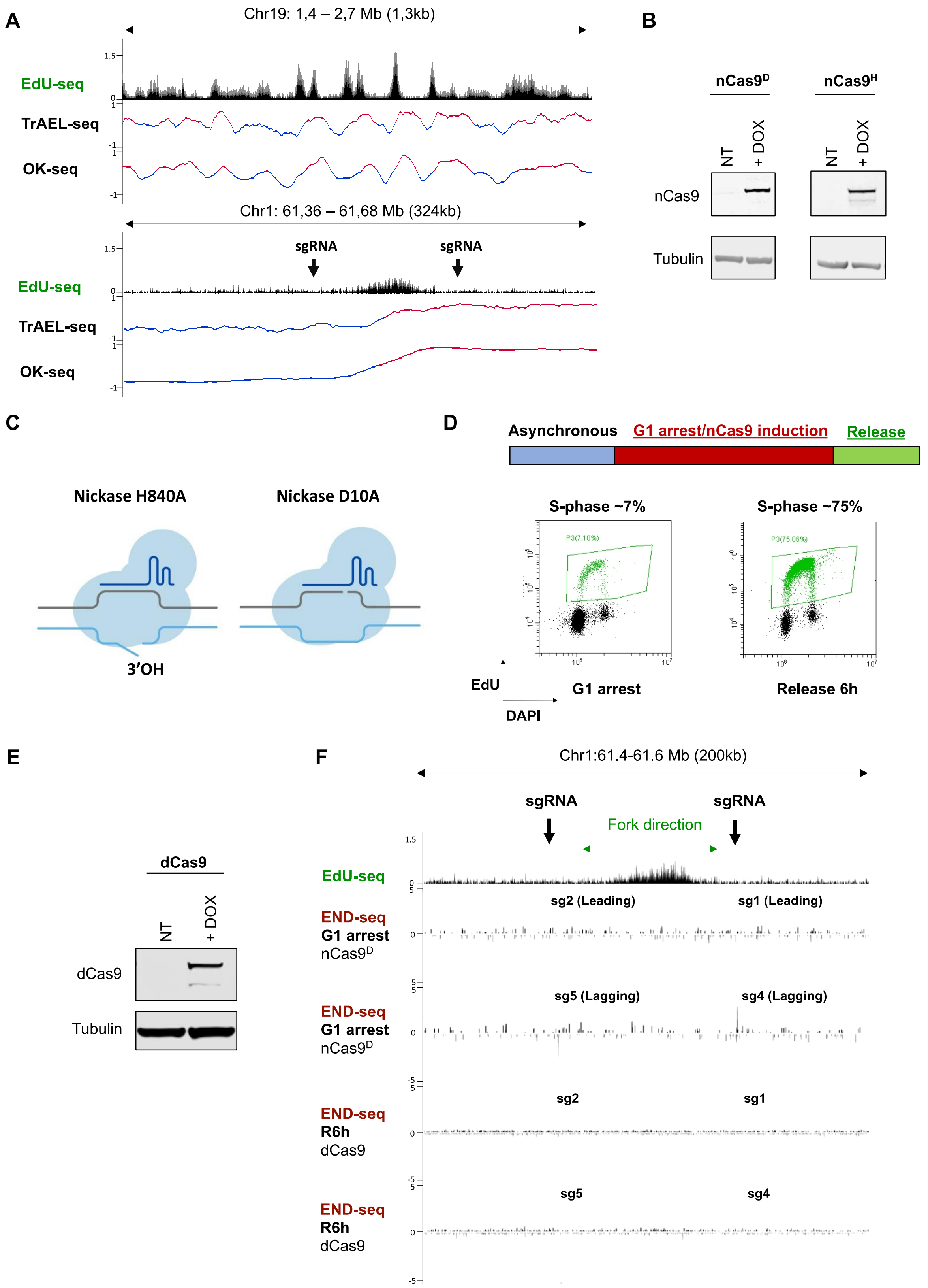

Fig. S1. Model system to study collapsed replication forks

(A) Genome browser screenshot displaying EdU-seq, TrAEL-seq, and OK-seq. EdU-seq was performed in MCF10A cells released from G1 arrest in the presence of 4μM APH, and TrAEL-seq was performed in asynchronous MCF10A cells. OK-seq was derived from (*25*). Black arrows indicate regions where the sgRNAs were designed to nick leading or lagging strands. **(B)** Western blot showing doxycycline inducible $nCas9^{D}$ and $nCas9^{H}$ expression in MCF10A cells. **(C)** Scheme of $nCas9^{H}$ (left) and $nCas9^{D}$ (right) generating a nick on the non-target strand and target strand, respectively. **(D)** Top panel: cell synchronization strategy; cells were arrested in G1 using 1μM Palbociclib, washed, and released in fresh media for 6h prior to collection. Bottom panel: cell cycle profile of MCF10A cells arrested in G1 and 6h after release from G1. Fluorescence-activated cell sorting (FACS) plots show EdU incorporation and DAPI. S-phase cells are highlighted in green. **(E)** Western Blot showing doxycycline-inducible dead Cas9 (dCas9) expression in MCF10A cells. **(F)** Genome browser screenshots displaying EdU-seq and END-seq profiles. Top panel depicts the positions of sgRNAs with respect to the targeted replication initiation zone, as mapped by EdU-seq in MCF10A cells. Second and third panels show END-seq profiles of G1 arrested cells expressing $nCas9^{D}$; sg1/sg2 and sg4/sg5 were designed for targeted nicking of the leading and lagging strand, respectively. The bottom two panels show END-seq profiles of dCas9-expressing cells following their release into S-phase; the same sgRNA pairs used to target $nCas9^{D}$ and dCas9.

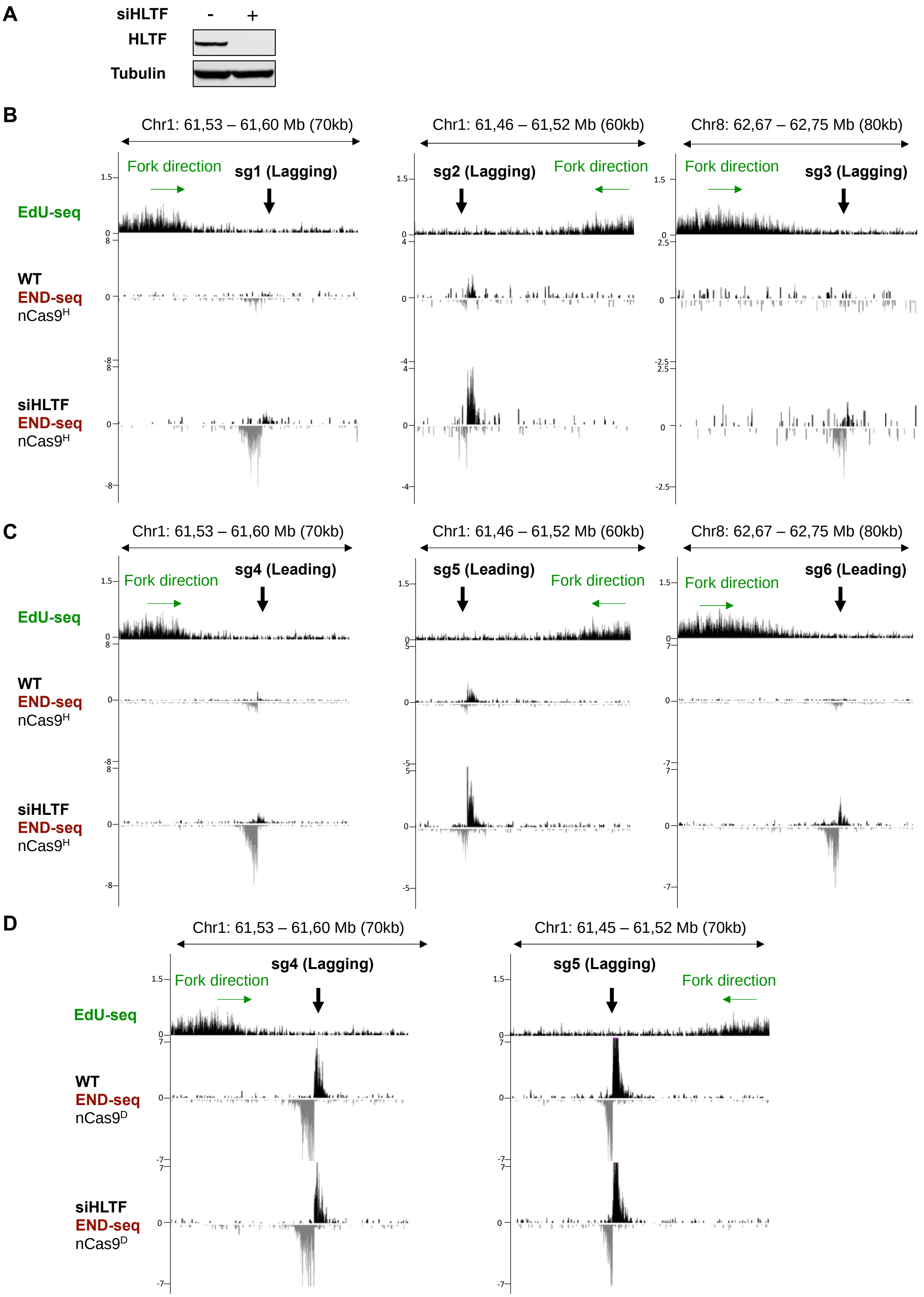

Fig. S2. HLTF limits nCas9H-induced fork collapse

(A) Western Blot analysis of HLTF knockdown by siRNA in MCF10A cells. **(B-D)** Genome browser screenshots displaying EdU-seq and END-seq profiles in MCF10A cells. Top panels depict the position of individual nicking sgRNA with respect to the targeted replication initiation zone, as mapped by EdU-seq. Middle and bottom panels, END-seq profiles of $nCas9^{H}$-expressing cells depleted or not for HLTF following lagging (**B**) or leading (**C**) strand fork collapse. **(D)** END-seq profiles of $nCas9^{D}$-expressing cells depleted or not for HLTF following lagging strand fork collapse. Cells were collected 6h after release from G1 arrest. Positive- and negative-strand END-seq reads in **B**-**D** are displayed in black and grey, respectively. Green arrows show replication fork direction.

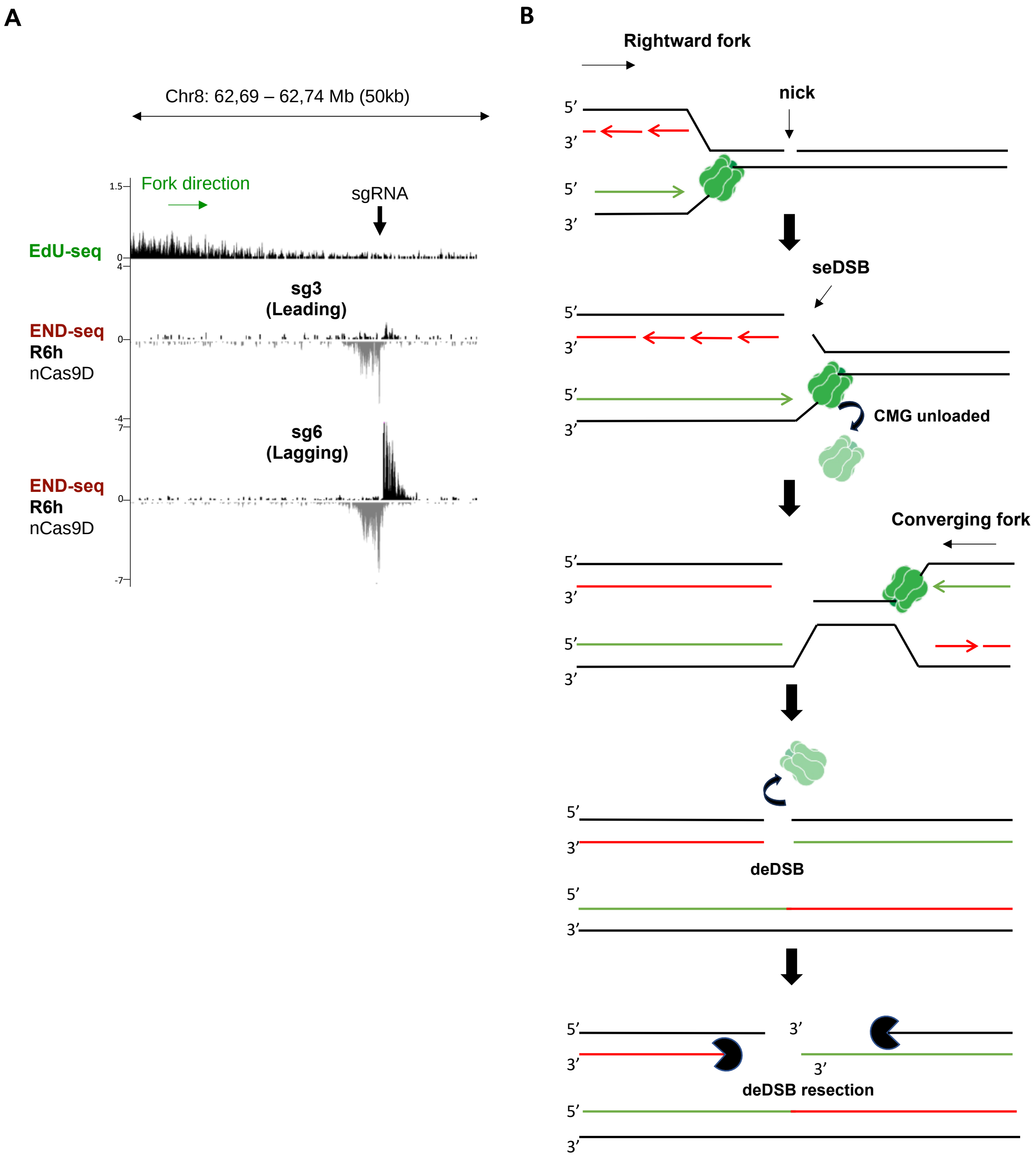

Fig. S3. Distinct DNA end structures at leading and lagging strand nick-induced DSBs

(A) Genome browser screenshots displaying EdU-seq and END-seq profiles as normalized read density (reads per million, RPM) in MCF10A cells. Top panel depict the position of individual nicking sgRNA with respect to the targeted replication initiation zone, as mapped by EdU-seq in MCF10A cells released from G1 arrest in the presence of 4μM APH. Lower panels show END-seq signals generated by fork collision with Cas9 D10A nickase ($nCas^{D}$)-induced nicks on the leading strand (middle) or lagging strand (bottom) in MCF10A cells 6h after release from G1 arrest. Positive- and negative-strand END-seq reads are displayed in black and grey, respectively. Green arrow shows replication fork direction. **(B)** Schematic in which a rightward fork collapses upon encountering a nick on the lagging strand. In this hypothetical model, collision with the converging fork induces the formation of a deDSB.

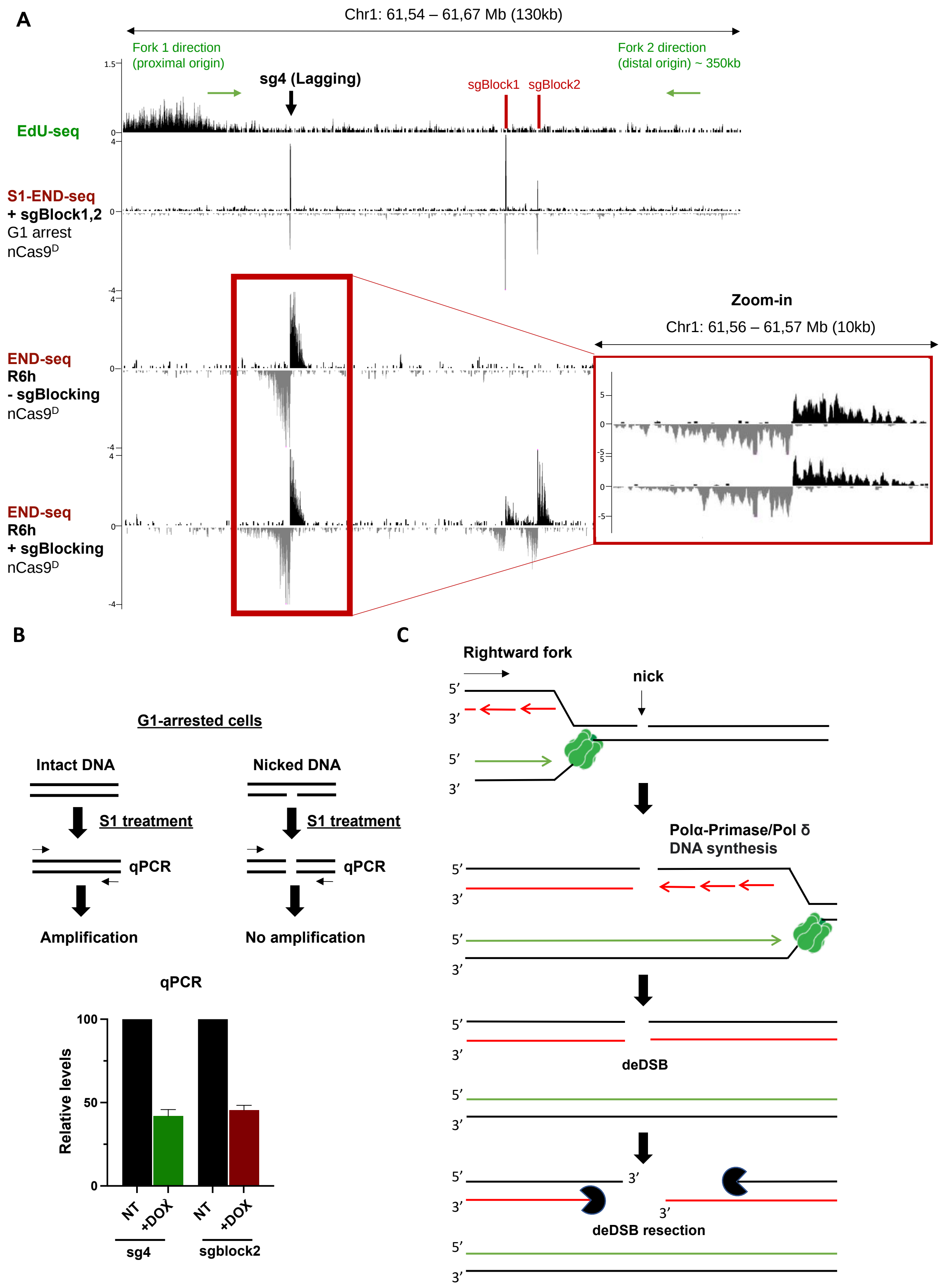

Fig. S4. Mechanism of deDSB formation at lagging strand collapsed forks

(A) Genome browser screenshots displaying EdU-seq and END-seq profiles in MCF10A cells. Top panel depicts the positions of the nicking and blocking sgRNAs with respect to the targeted replication initiation zone (fork 1- proximal origin) as well as the direction of the converging fork (fork 2- distal origin). Second panel shows S1-END-seq in G1 arrested MCF10A cells, confirming targeting by nCas^{D}/sg4, nCas^{D}/sgBlock1, and nCas^{D}/sgBlock2. Third and bottom panels show END-seq signals generated by nCas9^{D}/sg4 in MCF10A in the absence or presence of blocking sgRNAs 6h after release from G1. The zoom-in view highlights the DSB resection pattern. Positive- and negative-strand END-seq reads are displayed in black and grey, respectively. **(B)** qPCR assay to quantify nick frequency at sg4 and sgblock2 targeted regions. G1 arrested MCF10A cells were treated or not with Dox for 24h. The extracted genomic DNA was treated with recombinant S1 endonuclease to convert nicks into double-strand breaks and qPCR was performed using primers surrounding the nick sites. PCR amplification will occur when DNA molecules are intact but not broken (top panel). Graph shows amplification levels after nCas9^{D}/sg4 and nCas9^{D}/sgblock2 induction relative to NT samples. **(C)** Schematic working model in which a rightward fork bypasses a nick on the lagging strand leading to deDSB formation.

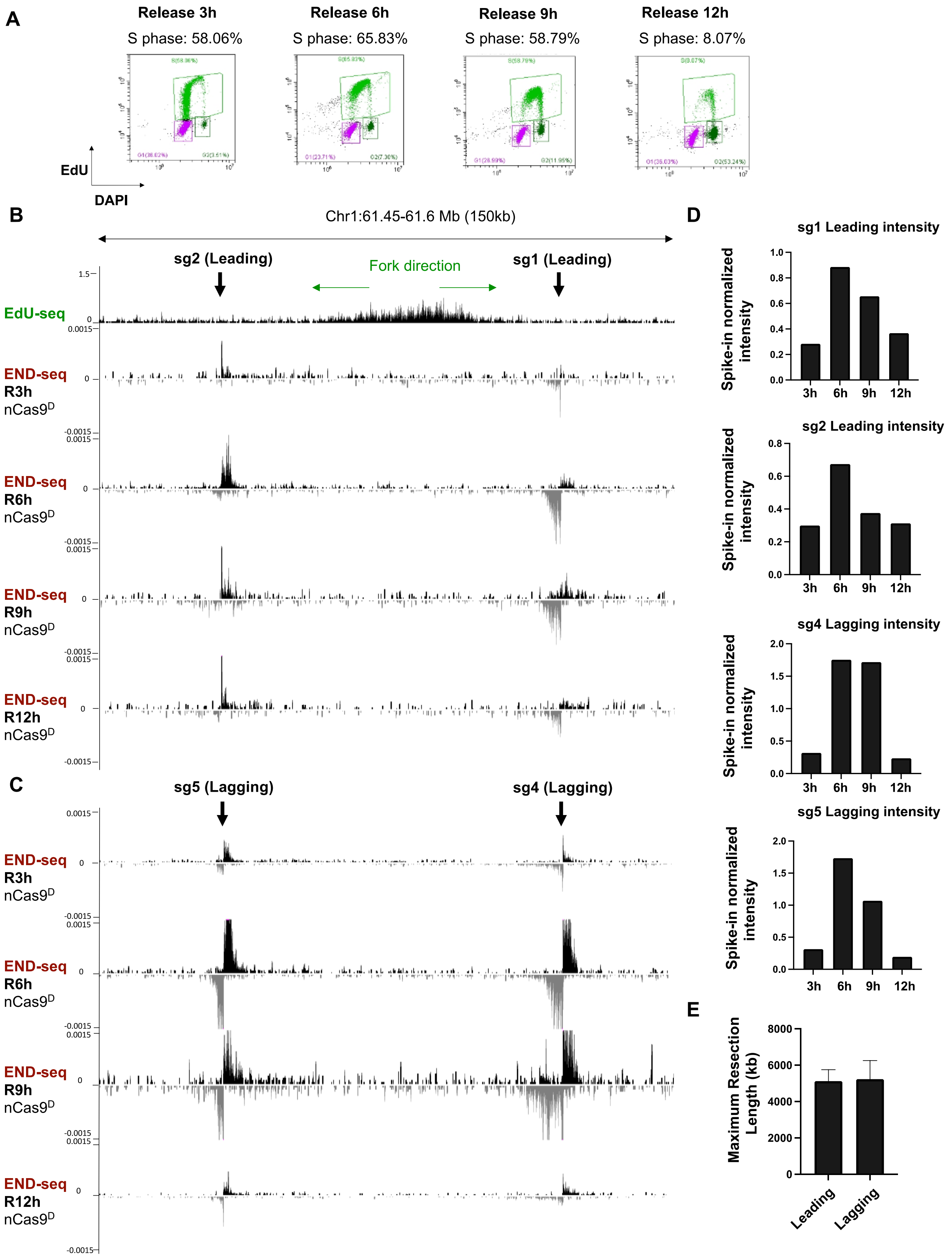

Fig. S5. Kinetics of DSB resolution at collapsed replication forks

(A) Cell cycle profiles of MCF10A cells at different timepoints after release from G1 arrest. Palbociclib was re-added 6h after release to prevent new cells from entering S phase. **(B)** Genome browser screenshots displaying EdU-seq and END-seq in MCF10A cells. Top panel depicts the positions of nicking sgRNAs with respect to the targeted replication initiation zone, as mapped by EdU-seq. Lower panels show END-seq signals generated by $nCas9^{D}$ at leading strand breaks at different timepoints after release from G1 arrest. **(C)** END-seq signals generated by $nCas9^{D}$ at lagging strand breaks at different timepoints after release from G1 arrest. (**D)** Quantification of the spike-in normalized intensity of END-seq signals from **B** and **C**.
(**E)** Maximum resection tract lengths of DNA ends generated by leading and lagging strand breaks. Positive- and negative-strand END-seq reads in **B** and **C** are displayed in black and grey, respectively. Green arrows show replication fork direction.

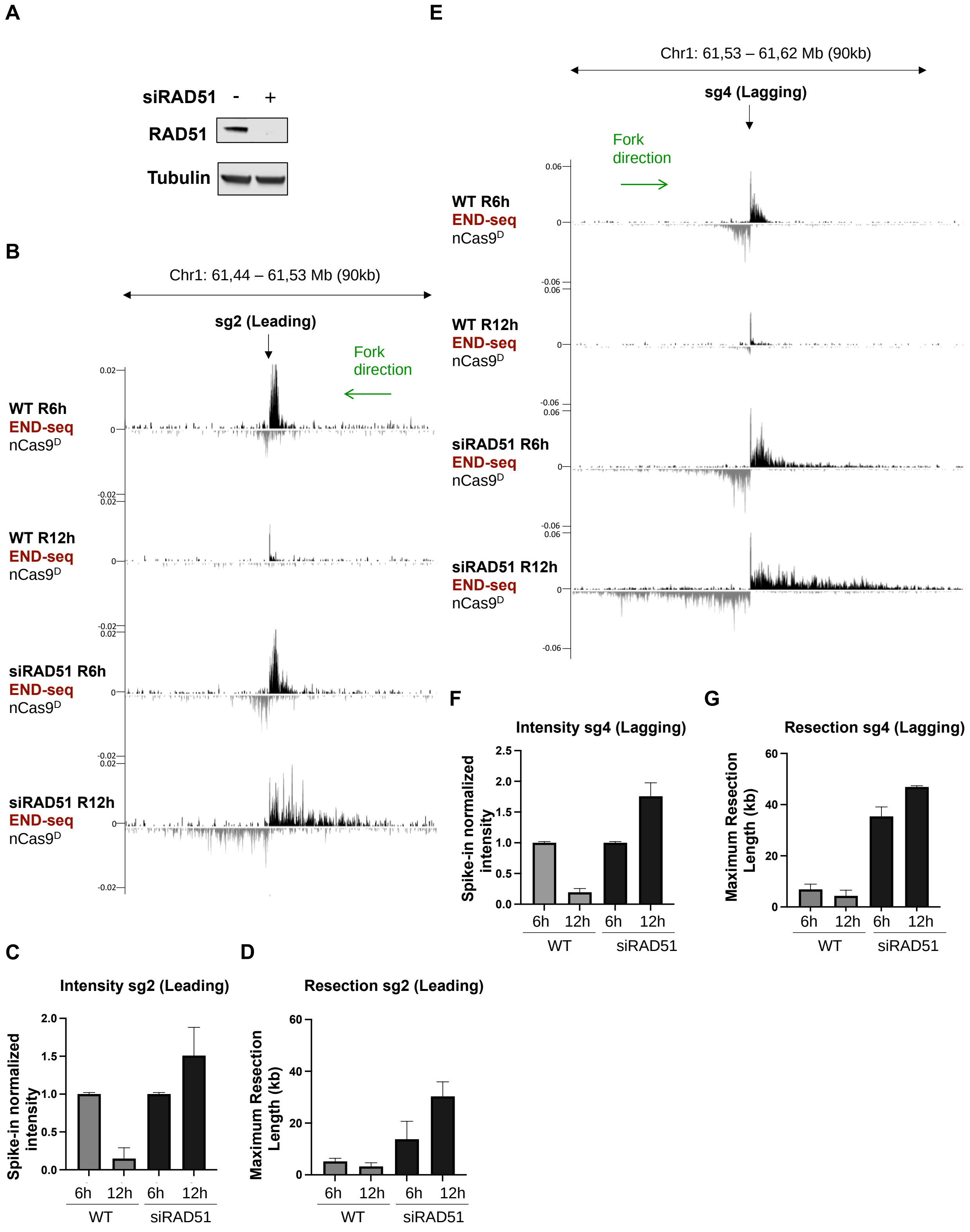

Fig. S6. RAD51 is essential for the resolution of DSBs at collapsed forks

(A) Western Blot analysis of RAD51 knockdown by siRNA in MCF10A cells. (**B)** Genome browser screenshots displaying END-seq signals at a leading strand fork collapse generated by nCas9^{D} in MCF10A treated or not with siRAD51. Cells were released for 6h or 12h from G1 arrest. Positive- and negative-strand END-seq reads are displayed in black and grey, respectively. Green arrow shows replication fork direction. **(C)** Spike-in normalized END-seq intensity signal at nCas9^{D}/sg4-induced collapsed forks quantified from three independent replicates. Signal at the R6h timepoint was normalized to 1, with intensity at R12h calculated relative to R6h. **(D)** Maximum resection lengths at sg2 collapsed forks quantified from three independent replicates. **(E)** Genome browser screenshots displaying END-seq signals at a lagging strand nick-induced DSB generated by nCas9^{D} in MCF10A treated or not with siRAD51. Cells were released for 6h or 12h from G1 arrest. Positive- and negative-strand END-seq reads are displayed in black and grey, respectively. Green arrow shows replication fork direction. **(F)** Spike-in normalized END-seq intensity signal at nCas9^{D}/sg4 collapsed forks quantified from three independent replicates. **(G)** Maximum resection lengths at nCas9^{D}/sg4 collapsed forks quantified from three independent replicates.

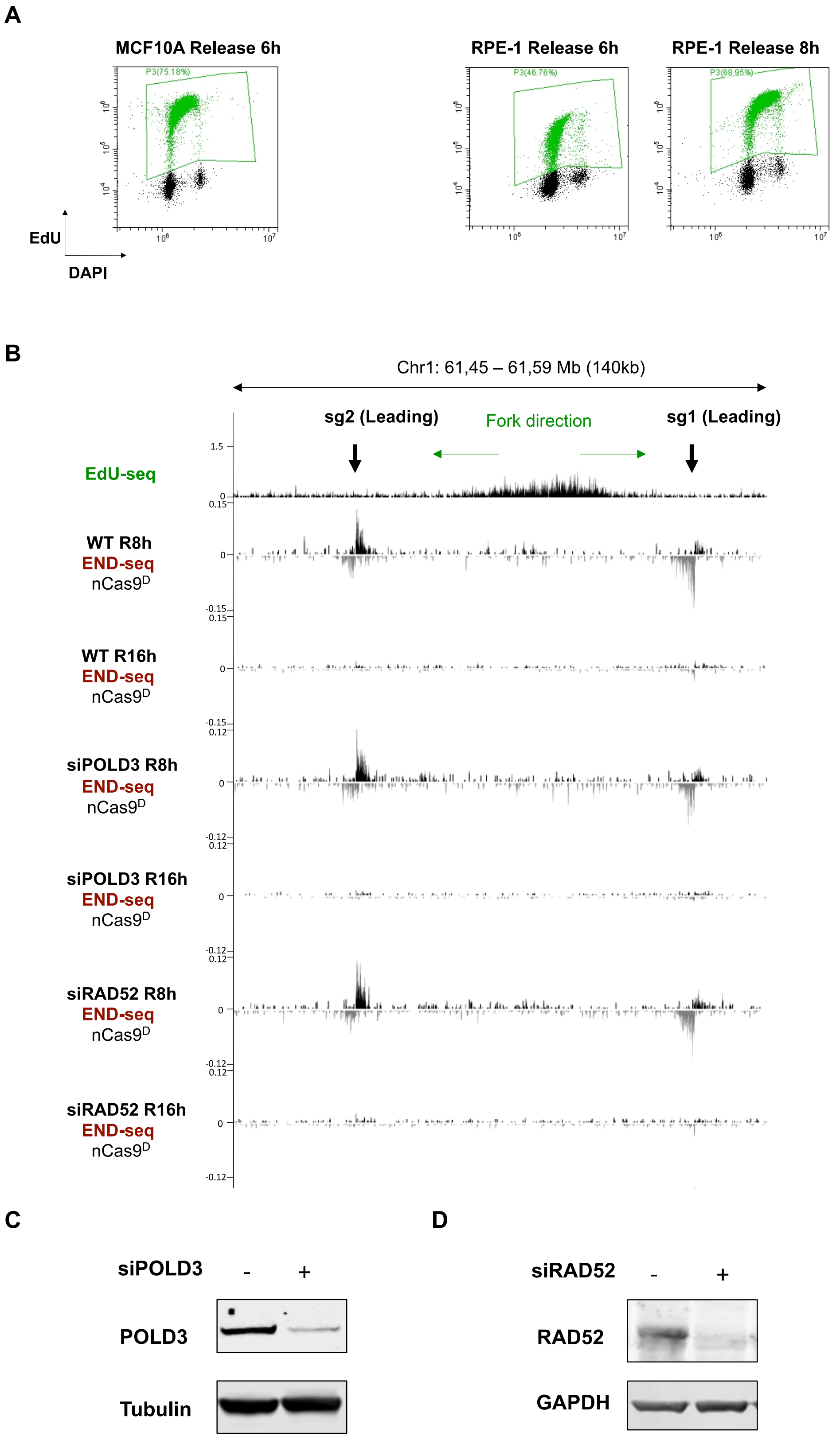

Fig. S7. Resolution of collapsed forks is independent of POLD3 and RAD52

(A) Cell cycle profiles of MCF10A and RPE-1 cells after release from G1 arrest. Note that release was slower in RPE-1 compared to MCF10A. **(B)** Genome browser screenshots displaying EdU-seq and END-seq profiles. Top panel depicts the positions of nicking sgRNAs with respect to the targeted replication initiation zone. Bottom panels show END-seq signals at leading strand collapsed forks generated by nCas9^{D} in RPE-1 cells treated or not with siRAD52 or siPOLD3; cells were collected 8h or 16h after release from G1 arrest. Positive- and negative-strand END-seq reads are displayed in black and grey, respectively. Green arrow shows replication fork direction. **(C, D)** Western Blot analysis of POLD3 (**C**) and RAD52 (**D**) after siRNA knockdown in RPE-1 cells.

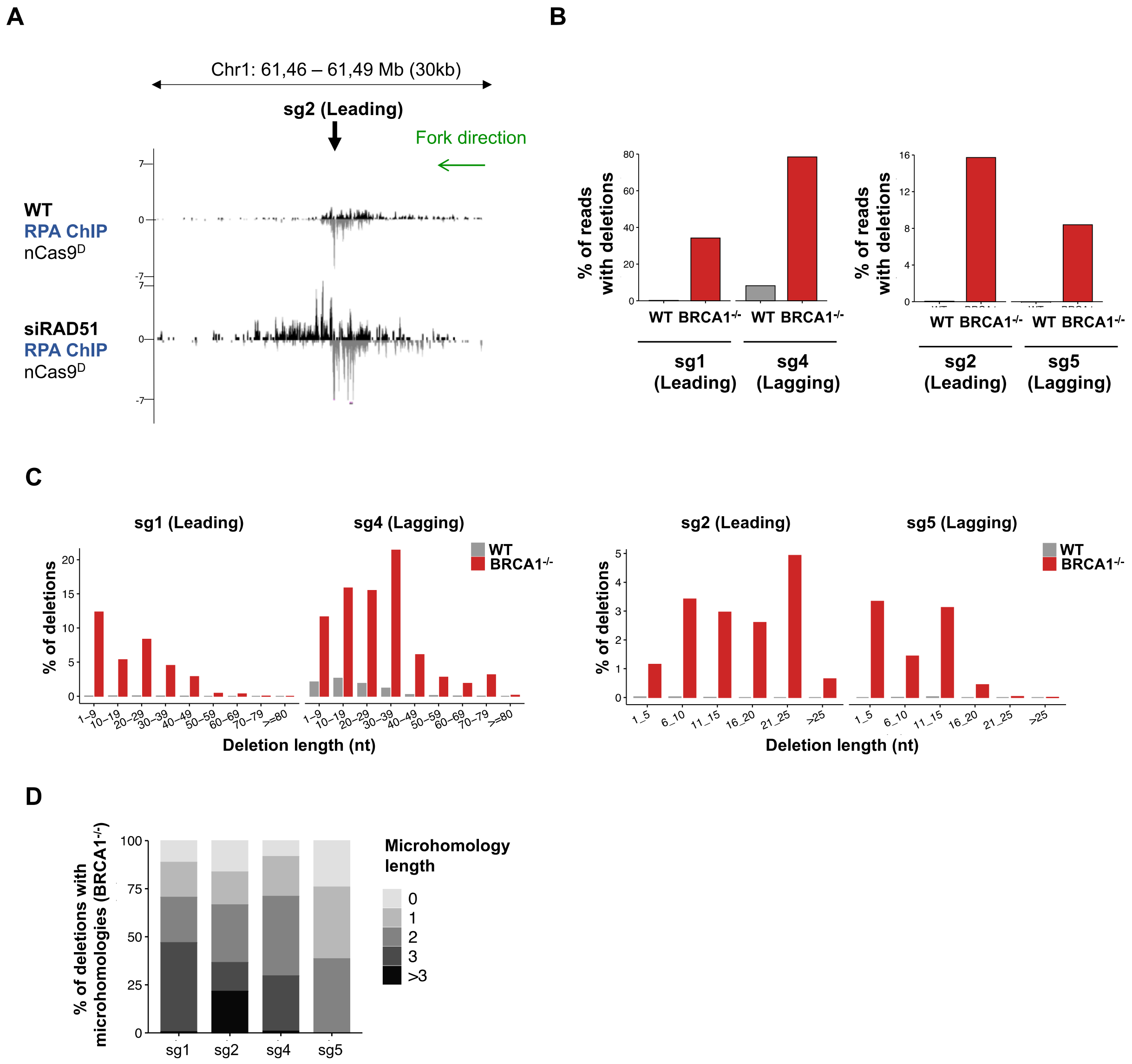

Fig. S8. Collapsed forks induce deletions in BRCA1-deficient cells

(A) Genome browser screenshots displaying RPA-bound ssDNA ChIP-seq profile at a leading strand collapsed fork generated by $nCas9^{D}$ in MCF10A cells treated or not with siRAD51. Positive- and negative-strand RPA-bound ssDNA ChIP-seq reads are displayed in black and grey, respectively. The green arrow shows the replication fork direction. **(B)** Percentage of total sequencing reads containing deletions surrounding $nCas9^{D}$ nick sites in WT (grey) and *BRCA1*$^{-/-}$ (red) RPE-1 cells. **(C)** Distribution of deletion lengths (Dox-induced minus non-treated) surrounding $nCas9^{D}$ nick sites in WT and *BRCA1*$^{-/-}$ RPE-1 cells. **(D)** Distribution of junctional microhomology lengths in *BRCA1*$^{-/-}$ RPE-1 cells.

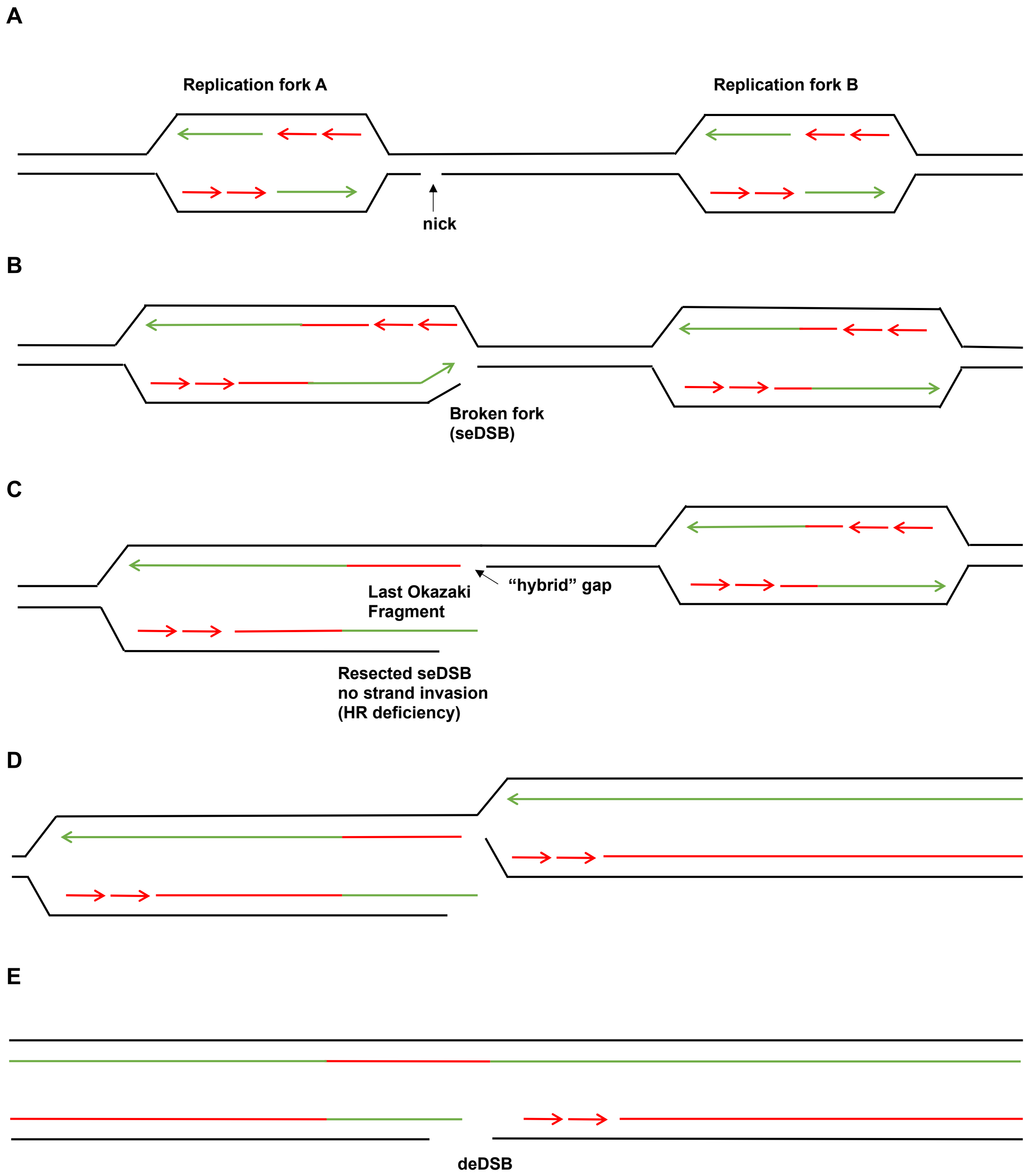

Fig. S9. Model for deDSB formation in the absence of strand invasion

When a rightward-moving fork collapses upon encountering a nick on the leading strand (**A)**, a seDSB is initially formed **(B)** and a “hybrid gap” is created between the last nascent Okazaki fragment and the parental strand (**C**). If the “hybrid” gap persists, a second DSB end is generated by a converging fork, leading to a deDSB (**D,E**).

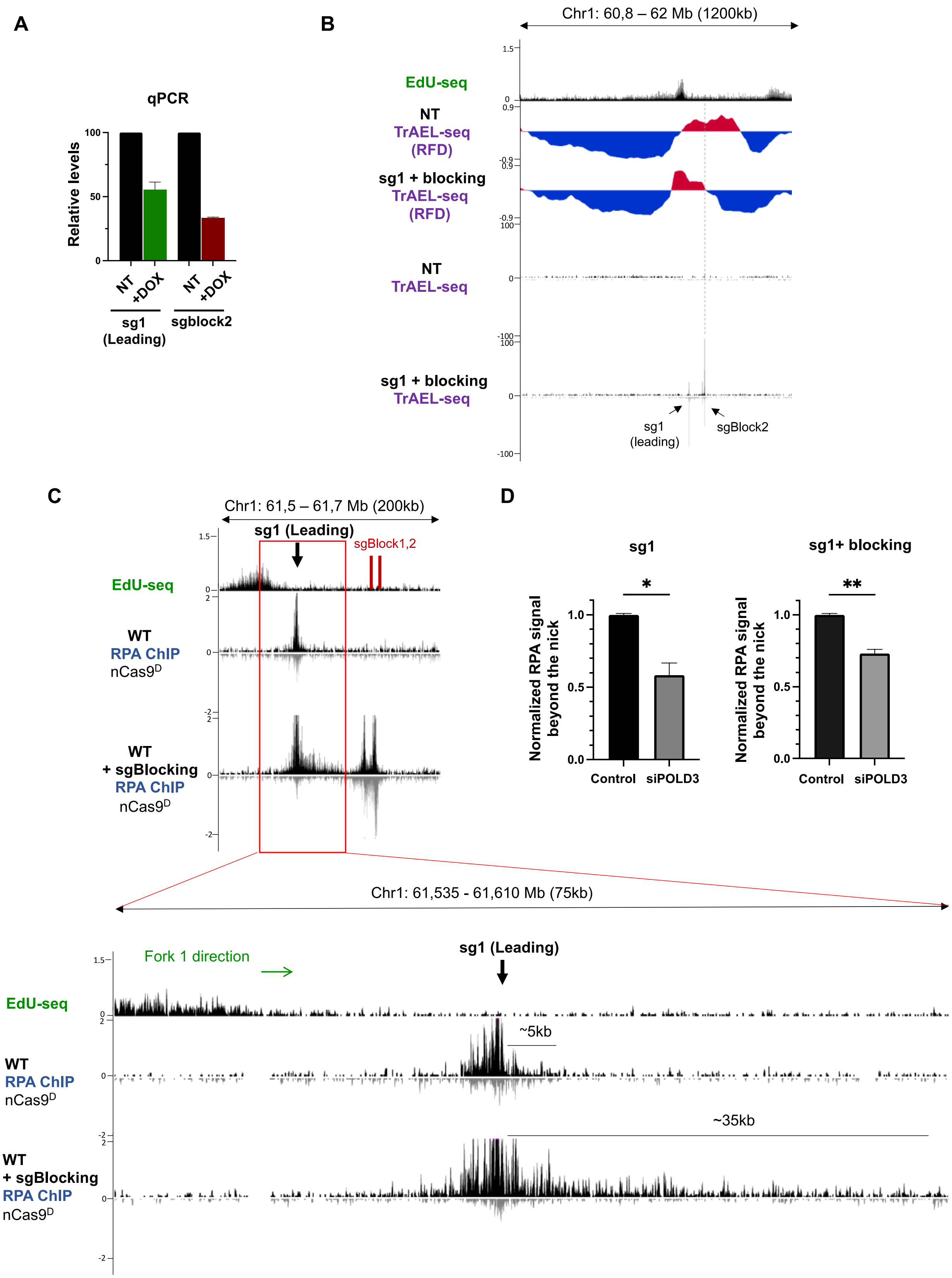

Fig. S10. Converging forks limit the extent of repair synthesis upon lead collapse.

(A) qPCR assay to quantify nick frequency at sg1- and sgblock2-targeted regions as described in fig. S4B. **(B)** Top panel**:** Genome browser screenshots displaying EdU-seq in MCF10A cells. Second and third panels: TrAEL-seq displaying replication fork directionality (RFD) calculated based on the ratio of plus and minus strand reads aggregated across 30kb windows (see methods). Fourth and fifth panel: TrAEL-seq genome browser screenshots as normalized reads (reads per million, RPM) at the nick sites. Experiments were performed in non-treated (NT) and doxycycline-treated MCF10A cells expressing sg1 and blocking sgRNAs. **(C)** Genome browser screenshots displaying EdU-seq and RPA-bound ssDNA ChIP-seq profiles in MCF10A cells. Top panel depicts the positions of the nicking and blocking sgRNAs with respect to the targeted replication initiation zone. Lower panels, RPA-bound ssDNA ChIP-seq signals at a leading strand collapsed fork generated by $nCas9^D$ in the absence or presence of blocking sgRNAs (sgBlock1 and sgBlock2) that impede converging forks. Samples were collected 6h after release from G1 arrest. Upon converging fork blockage, the RPA signal extends to the right side of the nick, indicative of more extensive strand invasion and D-loop migration as indicated in the zoom-in view. **(D)** Quantification of the RPA signal beyond the nick site from three independent replicates in MCF10A cells expressing sg1 or sg1+blocking guides treated or not with siPOLD3. Samples were collected 6h after release from G1 arrest. Statistical significance was determined by the unpaired t-test.

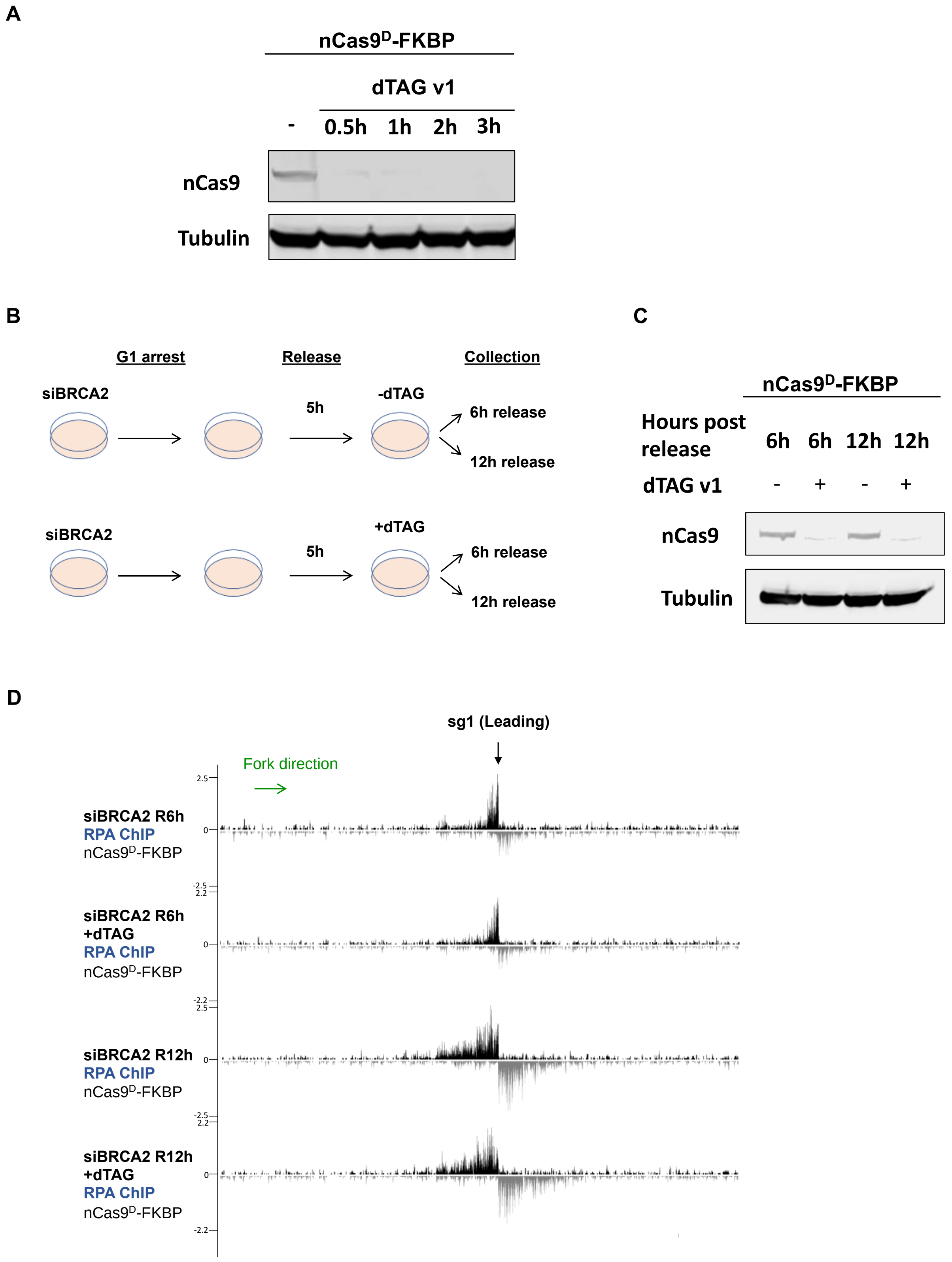

Fig. S11. deDSB generated from leading strand collapse in HR deficient cells are not due to

nCas9 re-nicking

(A) Western Blot analysis of nCas9^{D}-FKBP protein stability in MCF10A nCas9^{D} Ct-degron cells treated with dTAG-V1 for 0.5, 1, 2, and 3h. **(B)** Experimental design. MCF10A nCas9^{D} Ct-degron cells treated with siBRCA2 were arrested in G1 for 20h using 1μM Palbociclib. Doxycycline was added during the G1 arrest to induce the nickase. Thereafter, cells were washed and released from G1 arrest in fresh media. dTAG-V1 (0.5μM) was added 5h after release (+dTAG samples), and cells were collected at 6h and 12h after release (1h or 7h after dTAG addition, respectively). **(C)** Western Blot analysis of nCas9^{D}-FKBP protein in samples collected in **B**. **(D)** Genome browser screenshots displaying normalized RPA-bound ssDNA ChIP-seq signals at a leading strand fork collapse generated by nCas9^{D}-FKBP/sg1 in MCF10A nCas9^{D} Ct-degron cells. Positive- and negative-strand RPA-bound ssDNA ChIP-seq reads are displayed in black and grey, respectively. Green arrow shows replication fork direction.

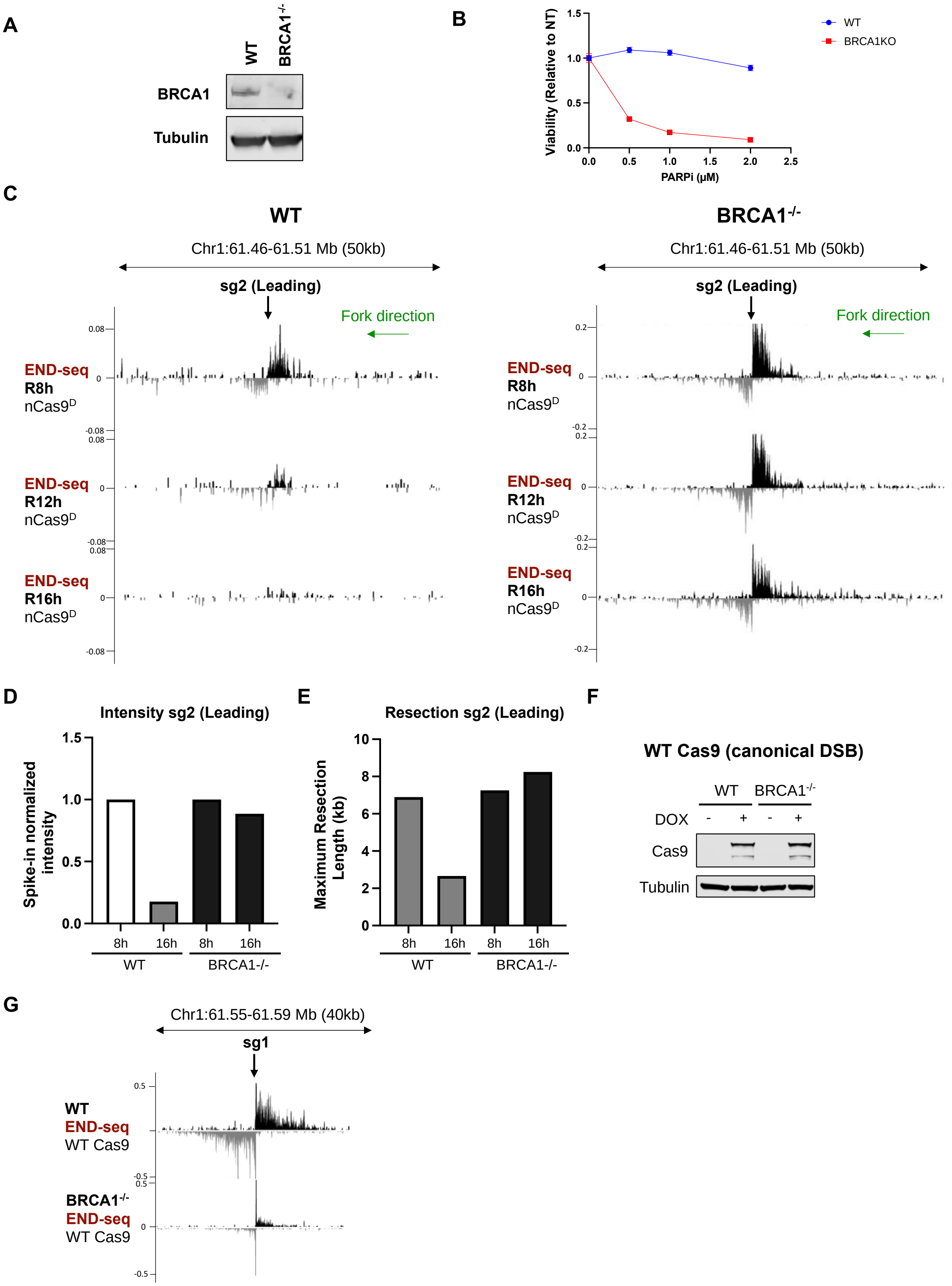

A
WT
BRCA1-/-
BRCA1
Tubulin
B
Viability (Relative to NT)
PARPi (μM)
WT
BRCA1KO
C
WT
BRCA1-/-
Chr1:61.46-61.51 Mb (50kb)
sg2 (Leading)
Fork direction
END-seq R8h nCas9D
END-seq R12h nCas9D
END-seq R16h nCas9D
D
Intensity sg2 (Leading)
Spike-in normalized intensity
8h 16h 8h 16h
WT BRCA1-/-
E
Resection sg2 (Leading)
Maximum Resection Length (kb)
8h 16h 8h 16h
WT BRCA1-/-
F
WT Cas9 (canonical DSB)
WT BRCA1-/-
DOX - + - +
Cas9
Tubulin
G
Chr1:61.55-61.59 Mb (40kb)
sg1
WT END-seq WT Cas9
BRCA1-/- END-seq WT Cas9

Fig. S12. BRCA1 is dispensable for resection but is essential for repair of nick-induced DSBs

(A) Western Blot analysis of BRCA1 in WT and *BRCA1*$^{-/-}$ RPE-1 cells. **(B)** Viability of WT and *BRCA1*$^{-/-}$ cells as measured by CellTiter-Glo 10 days after treatment with different concentrations of PARPi (Olaparib). Viability is normalized to non-treated controls. **(C)** Genome browser screenshots displaying END-seq signals at a leading strand collapse generated by nCas9^{D} in WT and *BRCA1*$^{-/-}$ cells collected 8h, 12h and 16h after release from G1 arrest. **(D)** Quantification of the spike-in normalized intensity of END-seq signals from three independent replicates. **(E)** Maximum resection tract lengths quantified from three independent replicates. **(F)** Western Blot analysis of Cas9 in WT and *BRCA1*$^{-/-}$ RPE-1 cells. **(G)** Genome browser screenshots displaying END-seq signals at a canonical DSB generated by Cas9 in WT and *BRCA1*$^{-/-}$ RPE-1 cells. Positive- and negative-strand END-seq reads in **C** and **G** are displayed in black and grey, respectively. Green arrow shows replication fork direction.

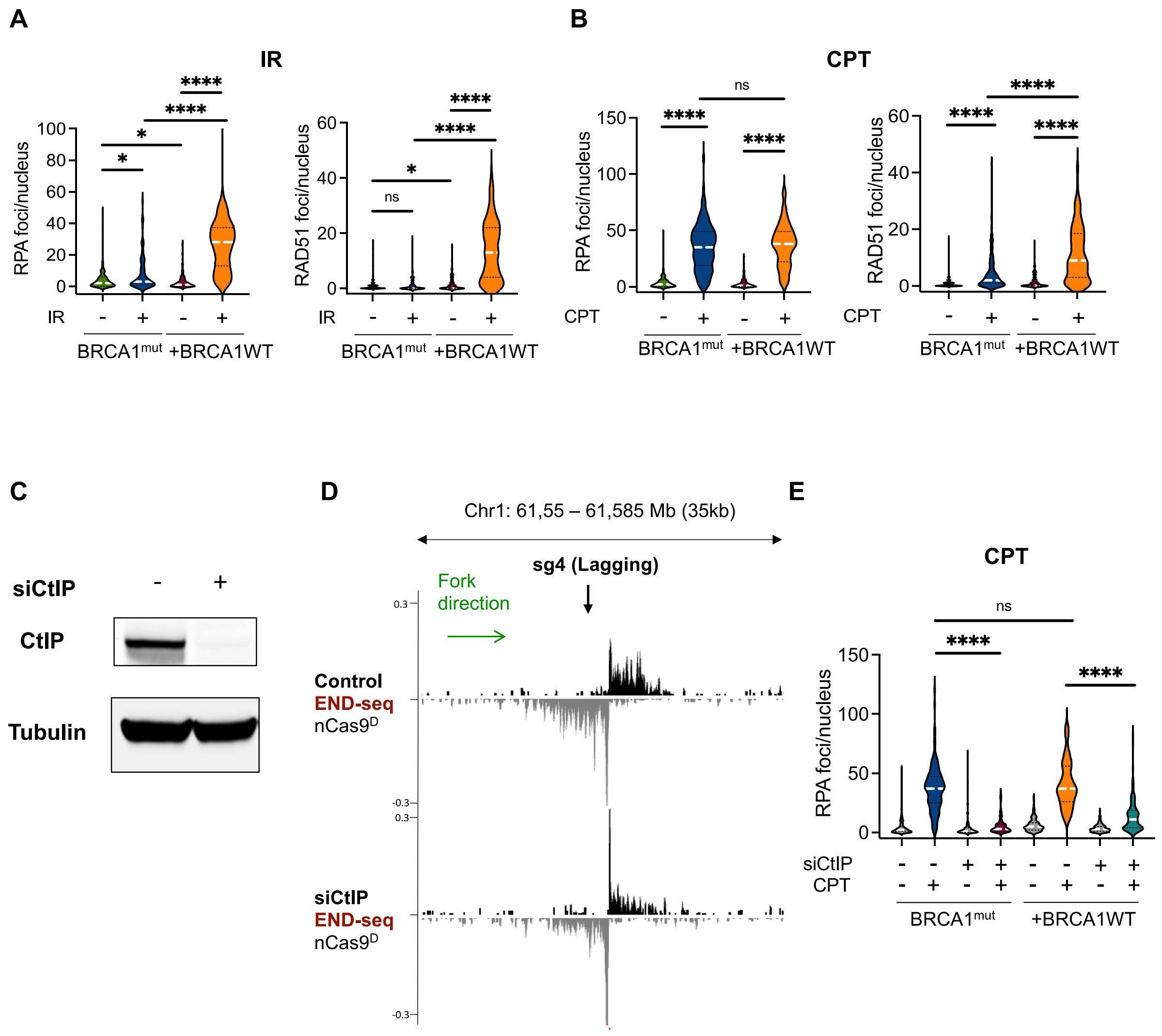

Fig. S13. Resection at collapsed forks is CtIP dependent.

(A, B) RPA and RAD51 foci per 5-ethynyl-2′-deoxyuridine (EdU) positive nucleus in MDA-MB-436 ($BRCA1^{mut}$) and MDA-MB-436 reconstituted with BRCA1WT (+BRCA1WT) cells measured 4 h after 5 Gy IR (**A**) or 1 h after 1μM Camptothecin (CPT) treatment (**B**). **(C)** Western Blot analysis of CtIP knockdown by siRNA in RPE-1 cells. (**D)** Genome browser screenshots displaying END–seq signals at a lagging strand nick-induced DSB generated by $nCas9^{D}$ in RPE-1 cells treated or not with siCtIP. Cells were collected 8h after release from G1 arrest. **(E)** RPA foci per EdU positive nucleus measured 1 h after 1μM CPT treatment in MDA-MB-436 ($BRCA1^{mut}$) and MDA-MB-436 reconstituted with BRCA1WT (+BRCA1WT) cells treated or not with siCtIP. Statistical significance in **A**, **B**, and **E** was determined by the Mann-Whitney test (**** $p<0.0001$, white dashed line represents the median, and black dashed lines represent the quartiles).

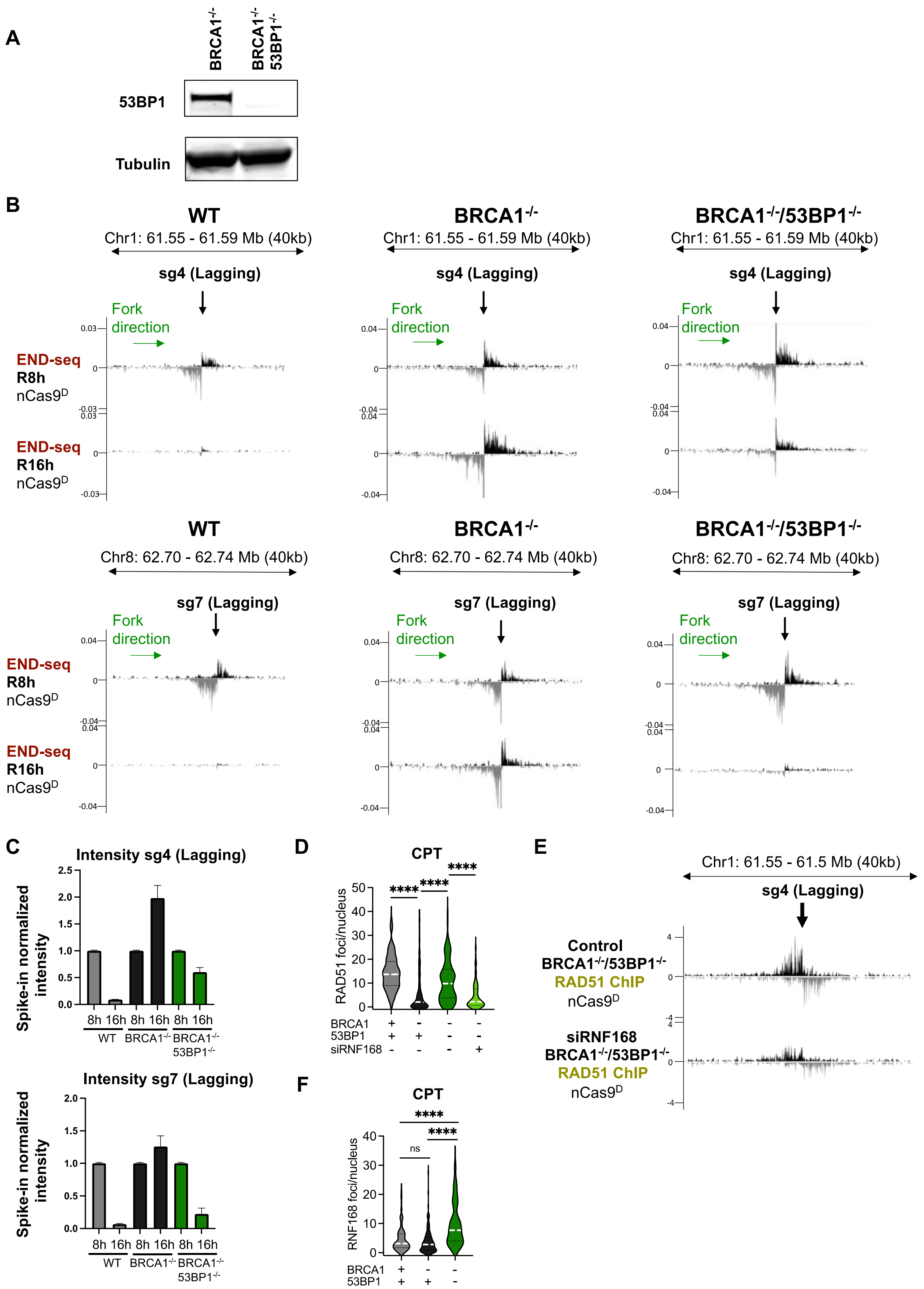

Fig. S14. 53BP1 depletion promotes RNF168-dependent RAD51 recruitment and repair of

collapsed forks in *BRCA1$^{-/-}$* cells

(A) Western Blot analysis of 53BP1 in *BRCA1$^{-/-}$* and *BRCA1$^{-/-}$53BP1$^{-/-}$* RPE-1 cells. **(B)** Genome browser screenshots displaying END-seq signals at lagging strand nick-induced DSB generated by nCas9^{D} in WT, *BRCA1$^{-/-}$*, and *BRCA1$^{-/-}$53BP1$^{-/-}$* RPE-1 cells collected 8h and 16h after release from G1 arrest. Positive- and negative-strand END-seq reads are displayed in black and grey, respectively. Green arrows show replication fork direction. **(C)** Quantification of the spike-in normalized intensity of END-seq signals from two independent replicates. **(D)** RAD51 foci per EdU positive nucleus measured 1 h after 1μM CPT treatment in WT, *BRCA1$^{-/-}$*, *BRCA1$^{-/-}$53BP1$^{-/-}$*, and *BRCA1$^{-/-}$53BP1$^{-/-}$* treated with siRNF168. Statistical significance was determined by the Mann-Whitney test (**** $p<0.0001$, white dashed line represents the median, and black dashed lines represent the quartiles). **(E)** Genome browser screenshots displaying RAD51 ChIP-seq signals at a lagging fork nick-induced DSB generated by nCas9^{D}/sg4 in *BRCA1$^{-/-}$53BP1$^{-/-}$* RPE-1 cells treated or not with siRNF168. Samples were collected after 8h release from G1 arrest. **(F)** RNF168 foci per EdU positive nucleus measured 1 h after 1μM CPT treatment in WT, *BRCA1$^{-/-}$*, and *BRCA1$^{-/-}$53BP1$^{-/-}$*. Statistical significance was determined by the Mann-Whitney test (**** $p<0.0001$, white dashed line represents the median, and black dashed lines represent the quartiles). Positive- and negative-strand END-seq reads in **B** and RAD51 ChIP-seq reads in **E** are displayed in black and grey, respectively.

Guide name	Guide sequence (without PAM)
sgLead1	5’ TACACTGTGGTATAAGGCAC 3’
sgLead2	5’ CTGCCCGCATGTCACGTTCA 3’
sgLead3	5’ TTTCACTGGCCCGTCAAGTG 3’
sgLag1	5’ CCACAGTGTAGAGTTTGCAC 3’
sgLag2	5’ GGTGCATGATGAGACAGCGC 3’
sgLag3	5’ GGGAGGGAGGATTAAGCTAA 3’
sgLag4	5’ TGGTGGCCTGCTAATTCGGG 3’
sgblock1	5’ CTGAGAGAGCCTATACTCCC 3’
sgblock2	5’ CTGGACTATGAGCTTCGTGA 3’
gR-Alu	5’ CAGGCGTGAGCCACCGCGCC 3’
AluGG	5’ CCTGTAGTCCCAGCTACTGG 3’

Table S1. List and sequence of sgRNAs.

Nick site	Orientation	Locus-specific sequence (5'-3')
sg1	Forward	ACCAAAAATATTGCAGTGGGGA
sg1	Reverse	AACTGGCCACACATTGACCT
sg2	Forward	AGAAAACCTGTCTGCCTTTCA
sg2	Reverse	GTAGCTTTCTTTCTCTACACACGC
sg4	Forward	ACCAAAAATATTGCAGTGGGGA
sg4	Reverse	AACTGGCCACACATTGACCT
sg5	Forward	TGGAAGAAGGGTGCATGATGA
sg5	Reverse	TCACTGAGTACCTATTCTTGGC

Table S2. List of locus-specific sequences used in the Amplicon-seq assays.

Nick site	Orientation	Locus-specific sequence (5'-3')
sg1	Forward	GGTCTCAATGCTGCAGAGAA
sg1	Reverse	GGATGACCCTATCCTTGGAA
sg2	Forward	GGTCTCAATGCTGCAGAGAA
sg2	Reverse	TAGGATGACCCTATCCTTGGAA
sgblock2	Forward	TACTCTGTGCCACAAACTGTAA
sgblock2	Reverse	GGGTCTCGTGCAGAAAGATAG
ACTB	Forward	AACTCCATCATGAAGTGTGACG
ACTB	Reverse	GATCCACATCTGCTGGAAGG

Table S3. Primers used in the qPCR assay.

References and Notes

1. E. V. Mirkin, S. M. Mirkin, Replication fork stalling at natural impediments. *Microbiol. Mol. Biol. Rev.* **71**, 13–35 (2007). doi:10.1128/MMBR.00030-06 Medline

2. D. Cortez, Replication-Coupled DNA Repair. *Mol. Cell* **74**, 866–876 (2019). doi:10.1016/j.molcel.2019.04.027 Medline

3. C. C. Chen, W. Feng, P. X. Lim, E. M. Kass, M. Jasin, Homology-Directed Repair and the Role of BRCA1, BRCA2, and Related Proteins in Genome Integrity and Cancer. *Annu. Rev. Cancer Biol.* **2**, 313–336 (2018). doi:10.1146/annurev-cancerbio-030617-050502 Medline

4. W. Liu, Y. Saito, J. Jackson, R. Bhowmick, M. T. Kanemaki, A. Vindigni, D. Cortez, RAD51 bypasses the CMG helicase to promote replication fork reversal. *Science* **380**, 382–387 (2023). doi:10.1126/science.add7328 Medline

5. R. Mayle, I. M. Campbell, C. R. Beck, Y. Yu, M. Wilson, C. A. Shaw, L. Bjergbaek, J. R. Lupski, G. Ira, Mus81 and converging forks limit the mutagenicity of replication fork breakage. *Science* **349**, 742–747 (2015). doi:10.1126/science.aaa8391 Medline

6. K. B. Vrtis, J. M. Dewar, G. Chistol, R. A. Wu, T. G. W. Graham, J. C. Walter, Single-strand DNA breaks cause replisome disassembly. *Mol. Cell* **81**, 1309–1318.e6 (2021). doi:10.1016/j.molcel.2020.12.039 Medline

7. M. M. Vilenchik, A. G. Knudson, Endogenous DNA double-strand breaks: Production, fidelity of repair, and induction of cancer. *Proc. Natl. Acad. Sci. U.S.A.* **100**, 12871–12876 (2003). doi:10.1073/pnas.2135498100 Medline

8. A. A. B. A. da Costa, D. Chowdhury, G. I. Shapiro, A. D. D'Andrea, P. A. Konstantinopoulos, Targeting replication stress in cancer therapy. *Nat. Rev. Drug Discov.* **22**, 38–58 (2023). doi:10.1038/s41573-022-00558-5 Medline

9. F. Liang, M. Han, P. J. Romanienko, M. Jasin, Homology-directed repair is a major double-strand break repair pathway in mammalian cells. *Proc. Natl. Acad. Sci. U.S.A.* **95**, 5172–5177 (1998). doi:10.1073/pnas.95.9.5172 Medline

10. P. Cejka, L. S. Symington, DNA End Resection: Mechanism and Control. *Annu. Rev. Genet.* **55**, 285–307 (2021). doi:10.1146/annurev-genet-071719-020312 Medline

11. R. M. Densham, J. R. Morris, Moving Mountains—The BRCA1 Promotion of DNA Resection. *Front. Mol. Biosci.* **6**, 79 (2019). doi:10.3389/fmolb.2019.00079 Medline

12. E. Callen, D. Zong, W. Wu, N. Wong, A. Stanlie, M. Ishikawa, R. Pavani, L. C. Dumitrache, A. K. Byrum, C. Mendez-Dorantes, P. Martinez, A. Canela, Y. Maman, A. Day, M. J. Kruhlak, M. A. Blasco, J. M. Stark, N. Mosammaparast, P. J. McKinnon, A. Nussenzweig, 53BP1 Enforces Distinct Pre- and Post-resection Blocks on Homologous Recombination. *Mol. Cell* **77**, 26–38.e7 (2020). doi:10.1016/j.molcel.2019.09.024 Medline

13. M. H. Yun, K. Hiom, CtIP-BRCA1 modulates the choice of DNA double-strand-break repair pathway throughout the cell cycle. *Nature* **459**, 460–463 (2009). doi:10.1038/nature07955 Medline

14. Z. Mirman, T. de Lange, 53BP1: A DSB escort. *Genes Dev.* **34**, 7–23 (2020). doi:10.1101/gad.333237.119 Medline

15. D. Setiaputra, D. Durocher, Shieldin - the protector of DNA ends. *EMBO Rep.* **20**, e47560 (2019). doi:10.15252/embr.201847560 Medline

16. R. Prakash, Y. Zhang, W. Feng, M. Jasin, Homologous recombination and human health: The roles of BRCA1, BRCA2, and associated proteins. *Cold Spring Harb. Perspect. Biol.* **7**, a016600 (2015). doi:10.1101/cshperspect.a016600 Medline

17. L. E. Vriend, R. Prakash, C.-C. Chen, F. Vanoli, F. Cavallo, Y. Zhang, M. Jasin, P. M. Krawczyk, Distinct genetic control of homologous recombination repair of Cas9-induced double-strand breaks, nicks and paired nicks. *Nucleic Acids Res.* **44**, 5204–5217 (2016). doi:10.1093/nar/gkw179 Medline

18. P. Bouwman, A. Aly, J. M. Escandell, M. Pieterse, J. Bartkova, H. van der Gulden, S. Hiddingh, M. Thanasoula, A. Kulkarni, Q. Yang, B. G. Haffty, J. Tommiska, C. Blomqvist, R. Drapkin, D. J. Adams, H. Nevanlinna, J. Bartek, M. Tarsounas, S. Ganesan, J. Jonkers, 53BP1 loss rescues BRCA1 deficiency and is associated with triple-negative and BRCA-mutated breast cancers. *Nat. Struct. Mol. Biol.* **17**, 688–695 (2010). doi:10.1038/nsmb.1831 Medline

19. S. F. Bunting, E. Callén, N. Wong, H.-T. Chen, F. Polato, A. Gunn, A. Bothmer, N. Feldhahn, O. Fernandez-Capetillo, L. Cao, X. Xu, C.-X. Deng, T. Finkel, M. Nussenzweig, J. M. Stark, A. Nussenzweig, 53BP1 inhibits homologous recombination in Brca1-deficient cells by blocking resection of DNA breaks. *Cell* **141**, 243–254 (2010). doi:10.1016/j.cell.2010.03.012 Medline

20. D. Zong, S. Adam, Y. Wang, H. Sasanuma, E. Callén, M. Murga, A. Day, M. J. Kruhlak, N. Wong, M. Munro, A. Ray Chaudhuri, B. Karim, B. Xia, S. Takeda, N. Johnson, D. Durocher, A. Nussenzweig, BRCA1 Haploinsufficiency Is Masked by RNF168-Mediated Chromatin Ubiquitylation. *Mol. Cell* **73**, 1267–1281.e7 (2019). doi:10.1016/j.molcel.2018.12.010 Medline

21. R. Belotserkovskaya, E. Raga Gil, N. Lawrence, R. Butler, G. Clifford, M. D. Wilson, S. P. Jackson, PALB2 chromatin recruitment restores homologous recombination in BRCA1-deficient cells depleted of 53BP1. *Nat. Commun.* **11**, 819 (2020). doi:10.1038/s41467-020-14563-y Medline

22. Y. L. Feng, Q. Liu, R.-D. Chen, S.-C. Liu, Z.-C. Huang, K.-M. Liu, X.-Y. Yang, A.-Y. Xie, DNA nicks induce mutational signatures associated with BRCA1 deficiency. *Nat. Commun.* **13**, 4285 (2022). doi:10.1038/s41467-022-32011-x Medline

23. N. Maizels, L. Davis, Initiation of homologous recombination at DNA nicks. *Nucleic Acids Res.* **46**, 6962–6973 (2018). doi:10.1093/nar/gky588 Medline

24. A. Tubbs, S. Sridharan, N. van Wietmarschen, Y. Maman, E. Callen, A. Stanlie, W. Wu, X. Wu, A. Day, N. Wong, M. Yin, A. Canela, H. Fu, C. Redon, S. C. Pruitt, Y. Jaszczyszyn, M. I. Aladjem, P. D. Aplan, O. Hyrien, A. Nussenzweig, Dual Roles of Poly(dA:dT) Tracts in Replication Initiation and Fork Collapse. *Cell* **174**, 1127–1142.e19 (2018). doi:10.1016/j.cell.2018.07.011 Medline

25. N. Petryk, M. Kahli, Y. d'Aubenton-Carafa, Y. Jaszczyszyn, Y. Shen, M. Silvain, C. Thermes, C.-L. Chen, O. Hyrien, Replication landscape of the human genome. *Nat. Commun.* **7**, 10208 (2016). doi:10.1038/ncomms10208 Medline

26. N. Kara, F. Krueger, P. Rugg-Gunn, J. Houseley, Genome-wide analysis of DNA replication and DNA double-strand breaks using TrAEL-seq. *PLOS Biol.* **19**, e3000886 (2021). doi:10.1371/journal.pbio.3000886 Medline

27. C. D. Richardson, G. J. Ray, M. A. DeWitt, G. L. Curie, J. E. Corn, Enhancing homology-directed genome editing by catalytically active and inactive CRISPR-Cas9 using asymmetric donor DNA. *Nat. Biotechnol.* **34**, 339–344 (2016). doi:10.1038/nbt.3481 Medline

28. A. Canela, S. Sridharan, N. Sciascia, A. Tubbs, P. Meltzer, B. P. Sleckman, A. Nussenzweig, DNA Breaks and End Resection Measured Genome-wide by End Sequencing. *Mol. Cell* **63**, 898–911 (2016). doi:10.1016/j.molcel.2016.06.034 Medline

29. A. Hishiki, K. Hara, Y. Ikegaya, H. Yokoyama, T. Shimizu, M. Sato, H. Hashimoto, Structure of a Novel DNA-binding Domain of Helicase-like Transcription Factor (HLTF) and Its Functional Implication in DNA Damage Tolerance. *J. Biol. Chem.* **290**, 13215–13223 (2015). doi:10.1074/jbc.M115.643643 Medline

30. A. C. Kile, D. A. Chavez, J. Bacal, S. Eldirany, D. M. Korzhnev, I. Bezsonova, B. F. Eichman, K. A. Cimprich, HLTF's Ancient HIRAN Domain Binds 3′ DNA Ends to Drive Replication Fork Reversal. *Mol. Cell* **58**, 1090–1100 (2015). doi:10.1016/j.molcel.2015.05.013 Medline

31. A. Bothmer, T. Phadke, L. A. Barrera, C. M. Margulies, C. S. Lee, F. Buquicchio, S. Moss, H. S. Abdulkerim, W. Selleck, H. Jayaram, V. E. Myer, C. Cotta-Ramusino, Characterization of the interplay between DNA repair and CRISPR/Cas9-induced DNA lesions at an endogenous locus. *Nat. Commun.* **8**, 13905 (2017). doi:10.1038/ncomms13905 Medline

32. X. Li, W. Chen, B. K. Martin, D. Calderon, C. Lee, J. Choi, F. M. Chardon, T. A. McDiarmid, R. M. Daza, H. Kim, J.-B. Lalanne, J. F. Nathans, D. S. Lee, J. Shendure, Chromatin context-dependent regulation and epigenetic manipulation of prime editing. *Cell* **187**, 2411–2427.e25 (2024). doi:10.1016/j.cell.2024.03.020 Medline

33. N. van Wietmarschen, S. Sridharan, W. J. Nathan, A. Tubbs, E. M. Chan, E. Callen, W. Wu, F. Belinky, V. Tripathi, N. Wong, K. Foster, J. Noorbakhsh, K. Garimella, A. Cruz-Migoni, J. A. Sommers, Y. Huang, A. A. Borah, J. T. Smith, J. Kalfon, N. Kesten, K. Fugger, R. L. Walker, E. Dolzhenko, M. A. Eberle, B. E. Hayward, K. Usdin, C. H. Freudenreich, R. M. Brosh Jr., S. C. West, P. J. McHugh, P. S. Meltzer, A. J. Bass, A. Nussenzweig, Repeat expansions confer WRN dependence in microsatellite-unstable cancers. *Nature* **586**, 292–298 (2020). doi:10.1038/s41586-020-2769-8 Medline

34. K. P. Jakobsen, K. O. Nielsen, K. V. Løvschal, M. Rødgaard, A. H. Andersen, L. Bjergbæk, Minimal Resection Takes Place during Break-Induced Replication Repair of Collapsed Replication Forks and Is Controlled by Strand Invasion. *Cell Rep.* **26**, 836–844.e3 (2019). doi:10.1016/j.celrep.2018.12.108 Medline

35. S. K. Sotiriou, I. Kamileri, N. Lugli, K. Evangelou, C. Da-Ré, F. Huber, L. Padayachy, S. Tardy, N. L. Nicati, S. Barriot, F. Ochs, C. Lukas, J. Lukas, V. G. Gorgoulis, L.

Scapozza, T. D. Halazonetis, Mammalian RAD52 Functions in Break-Induced Replication Repair of Collapsed DNA Replication Forks. *Mol. Cell* **64**, 1127–1134 (2016). doi:10.1016/j.molcel.2016.10.038 Medline

36. L. Costantino, S. K. Sotiriou, J. K. Rantala, S. Magin, E. Mladenov, T. Helleday, J. E. Haber, G. Iliakis, O. P. Kallioniemi, T. D. Halazonetis, Break-induced replication repair of damaged forks induces genomic duplications in human cells. *Science* **343**, 88–91 (2014). doi:10.1126/science.1243211 Medline

37. P. P. Khil, F. Smagulova, K. M. Brick, R. D. Camerini-Otero, G. V. Petukhova, Sensitive mapping of recombination hotspots using sequencing-based detection of ssDNA. *Genome Res.* **22**, 957–965 (2012). doi:10.1101/gr.130583.111 Medline

38. J. L. Sneeden, S. M. Grossi, I. Tappin, J. Hurwitz, W. D. Heyer, Reconstitution of recombination-associated DNA synthesis with human proteins. *Nucleic Acids Res.* **41**, 4913–4925 (2013). doi:10.1093/nar/gkt192 Medline

39. E. Johansson, J. F. X. Diffley, Unchecked nick ligation can promote localized genome re-replication. *Curr. Biol.* **31**, R710–R711 (2021). doi:10.1016/j.cub.2021.03.043 Medline

40. A. Cruz-García, A. López-Saavedra, P. Huertas, BRCA1 accelerates CtIP-mediated DNA-end resection. *Cell Rep.* **9**, 451–459 (2014). doi:10.1016/j.celrep.2014.08.076 Medline

41. J. M. Stark, A. J. Pierce, J. Oh, A. Pastink, M. Jasin, Genetic steps of mammalian homologous repair with distinct mutagenic consequences. *Mol. Cell. Biol.* **24**, 9305–9316 (2004). doi:10.1128/MCB.24.21.9305-9316.2004 Medline

42. F. Zhang, Q. Fan, K. Ren, P. R. Andreassen, PALB2 functionally connects the breast cancer susceptibility proteins BRCA1 and BRCA2. *Mol. Cancer Res.* **7**, 1110–1118 (2009). doi:10.1158/1541-7786.MCR-09-0123 Medline

43. F. Zhang, J. Ma, J. Wu, L. Ye, H. Cai, B. Xia, X. Yu, PALB2 links BRCA1 and BRCA2 in the DNA-damage response. *Curr. Biol.* **19**, 524–529 (2009). doi:10.1016/j.cub.2009.02.018 Medline

44. W. Zhao, J. B. Steinfeld, F. Liang, X. Chen, D. G. Maranon, C. Jian Ma, Y. Kwon, T. Rao, W. Wang, C. Sheng, X. Song, Y. Deng, J. Jimenez-Sainz, L. Lu, R. B. Jensen, Y. Xiong, G. M. Kupfer, C. Wiese, E. C. Greene, P. Sung, BRCA1-BARD1 promotes RAD51-mediated homologous DNA pairing. *Nature* **550**, 360–365 (2017). doi:10.1038/nature24060 Medline

45. N. Johnson, S. F. Johnson, W. Yao, Y.-C. Li, Y.-E. Choi, A. J. Bernhardy, Y. Wang, M. Capelletti, K. A. Sarosiek, L. A. Moreau, D. Chowdhury, A. Wickramanayake, M. I. Harrell, J. F. Liu, A. D. D'Andrea, A. Miron, E. M. Swisher, G. I. Shapiro, Stabilization of mutant BRCA1 protein confers PARP inhibitor and platinum resistance. *Proc. Natl. Acad. Sci. U.S.A.* **110**, 17041–17046 (2013). doi:10.1073/pnas.1305170110 Medline

46. L. C. Gowen, B. L. Johnson, A. M. Latour, K. K. Sulik, B. H. Koller, *Brca1* deficiency results in early embryonic lethality characterized by neuroepithelial abnormalities. *Nat. Genet.* **12**, 191–194 (1996). doi:10.1038/ng0296-191 Medline

47. R. Hakem, J. L. de la Pompa, C. Sirard, R. Mo, M. Woo, A. Hakem, A. Wakeham, J. Potter, A. Reitmair, F. Billia, E. Firpo, C. C. Hui, J. Roberts, J. Rossant, T. W. Mak, The tumor

suppressor gene *Brca1* is required for embryonic cellular proliferation in the mouse. *Cell* **85**, 1009–1023 (1996). doi:10.1016/S0092-8674(00)81302-1 Medline

48. T. Ludwig, D. L. Chapman, V. E. Papaioannou, A. Efstratiadis, Targeted mutations of breast cancer susceptibility gene homologs in mice: Lethal phenotypes of Brca1, Brca2, Brca1/Brca2, Brca1/p53, and Brca2/p53 nullizygous embryos. *Genes Dev.* **11**, 1226–1241 (1997). doi:10.1101/gad.11.10.1226 Medline

49. E. Callen, M. Di Virgilio, M. J. Kruhlak, M. Nieto-Soler, N. Wong, H.-T. Chen, R. B. Faryabi, F. Polato, M. Santos, L. M. Starnes, D. R. Wesemann, J.-E. Lee, A. Tubbs, B. P. Sleckman, J. A. Daniel, K. Ge, F. W. Alt, O. Fernandez-Capetillo, M. C. Nussenzweig, A. Nussenzweig, 53BP1 mediates productive and mutagenic DNA repair through distinct phosphoprotein interactions. *Cell* **153**, 1266–1280 (2013). doi:10.1016/j.cell.2013.05.023 Medline

50. M. S. Luijsterburg, D. Typas, M.-C. Caron, W. W. Wiegant, D. van den Heuvel, R. A. Boonen, A. M. Couturier, L. H. Mullenders, J.-Y. Masson, H. van Attikum, A PALB2-interacting domain in RNF168 couples homologous recombination to DNA break-induced chromatin ubiquitylation. *eLife* **6**, e20922 (2017). doi:10.7554/eLife.20922 Medline

51. M. Gatti, S. Pinato, E. Maspero, P. Soffientini, S. Polo, L. Penengo, A novel ubiquitin mark at the N-terminal tail of histone H2As targeted by RNF168 ubiquitin ligase. *Cell Cycle* **11**, 2538–2544 (2012). doi:10.4161/cc.20919 Medline

52. F. Mattiroli, J. H. A. Vissers, W. J. van Dijk, P. Ikpa, E. Citterio, W. Vermeulen, J. A. Marteijn, T. K. Sixma, RNF168 ubiquitinates K13-15 on H2A/H2AX to drive DNA damage signaling. *Cell* **150**, 1182–1195 (2012). doi:10.1016/j.cell.2012.08.005 Medline

53. S. Panier, Y. Ichijima, A. Fradet-Turcotte, C. C. Y. Leung, L. Kaustov, C. H. Arrowsmith, D. Durocher, Tandem protein interaction modules organize the ubiquitin-dependent response to DNA double-strand breaks. *Mol. Cell* **47**, 383–395 (2012). doi:10.1016/j.molcel.2012.05.045 Medline

54. A. Fradet-Turcotte, M. D. Canny, C. Escribano-Díaz, A. Orthwein, C. C. Y. Leung, H. Huang, M.-C. Landry, J. Kitevski-LeBlanc, S. M. Noordermeer, F. Sicheri, D. Durocher, 53BP1 is a reader of the DNA-damage-induced H2A Lys 15 ubiquitin mark. *Nature* **499**, 50–54 (2013). doi:10.1038/nature12318 Medline

55. K. Nakamura, G. Kustatscher, C. Alabert, M. Hödl, I. Forne, M. Völker-Albert, S. Satpathy, T. E. Beyer, N. Mailand, C. Choudhary, A. Imhof, J. Rappsilber, A. Groth, Proteome dynamics at broken replication forks reveal a distinct ATM-directed repair response suppressing DNA double-strand break ubiquitination. *Mol. Cell* **81**, 1084–1099.e6 (2021). doi:10.1016/j.molcel.2020.12.025 Medline

56. C. J. Smith, O. Castanon, K. Said, V. Volf, P. Khoshakhlagh, A. Hornick, R. Ferreira, C.-T. Wu, M. Güell, S. Garg, A. H. M. Ng, H. Myllykallio, G. M. Church, Enabling large-scale genome editing at repetitive elements by reducing DNA nicking. *Nucleic Acids Res.* **48**, 5183–5195 (2020). doi:10.1093/nar/gkaa239 Medline

57. R. S. Zou, A. Marin-Gonzalez, Y. Liu, H. B. Liu, L. Shen, R. K. Dveirin, J. X. J. Luo, R. Kalhor, T. Ha, Massively parallel genomic perturbations with multi-target CRISPR

interrogates Cas9 activity and DNA repair at endogenous sites. *Nat. Cell Biol.* **24**, 1433–1444 (2022). doi:10.1038/s41556-022-00975-z Medline

58. H. E. Bryant, N. Schultz, H. D. Thomas, K. M. Parker, D. Flower, E. Lopez, S. Kyle, M. Meuth, N. J. Curtin, T. Helleday, Specific killing of BRCA2-deficient tumours with inhibitors of poly(ADP-ribose) polymerase. *Nature* **434**, 913–917 (2005). doi:10.1038/nature03443 Medline

59. H. Farmer, N. McCabe, C. J. Lord, A. N. J. Tutt, D. A. Johnson, T. B. Richardson, M. Santarosa, K. J. Dillon, I. Hickson, C. Knights, N. M. B. Martin, S. P. Jackson, G. C. M. Smith, A. Ashworth, Targeting the DNA repair defect in BRCA mutant cells as a therapeutic strategy. *Nature* **434**, 917–921 (2005). doi:10.1038/nature03445 Medline

60. K. W. Caldecott, Causes and consequences of DNA single-strand breaks. *Trends Biochem. Sci.* **49**, 68–78 (2024). doi:10.1016/j.tibs.2023.11.001 Medline

61. M. Fiumara, S. Ferrari, A. Omer-Javed, S. Beretta, L. Albano, D. Canarutto, A. Varesi, C. Gaddoni, C. Brombin, F. Cugnata, E. Zonari, M. M. Naldini, M. Barcella, B. Gentner, I. Merelli, L. Naldini, Genotoxic effects of base and prime editing in human hematopoietic stem cells. *Nat. Biotechnol.* (2023). doi:10.1038/s41587-023-01915-4 Medline

62. M. E. Huang, Y. Qin, Y. Shang, Q. Hao, C. Zhan, C. Lian, S. Luo, L. D. Liu, S. Zhang, Y. Zhang, Y. Wo, N. Li, S. Wu, T. Gui, B. Wang, Y. Luo, Y. Cai, X. Liu, Z. Xu, P. Dai, S. Li, L. Zhang, J. Dong, J. Wang, X. Zheng, Y. Xu, Y. Sun, W. Wu, L.-S. Yeap, F.-L. Meng, C-to-G editing generates double-strand breaks causing deletion, transversion and translocation. *Nat. Cell Biol.* **26**, 294–304 (2024). doi:10.1038/s41556-023-01342-2 Medline

63. Y. Pommier, A. Nussenzweig, S. Takeda, C. Austin, Human topoisomerases and their roles in genome stability and organization. *Nat. Rev. Mol. Cell Biol.* **23**, 407–427 (2022). doi:10.1038/s41580-022-00452-3 Medline

64. J. R. Brickner, J. L. Garzon, K. A. Cimprich, Walking a tightrope: The complex balancing act of R-loops in genome stability. *Mol. Cell* **82**, 2267–2297 (2022). doi:10.1016/j.molcel.2022.04.014 Medline

65. E. Henckaerts, N. Dutheil, N. Zeltner, S. Kattman, E. Kohlbrenner, P. Ward, N. Clément, P. Rebollo, M. Kennedy, G. M. Keller, R. M. Linden, Site-specific integration of adeno-associated virus involves partial duplication of the target locus. *Proc. Natl. Acad. Sci. U.S.A.* **106**, 7571–7576 (2009). doi:10.1073/pnas.0806821106 Medline

66. R. Elango, N. Nilavar, A. G. Li, E. E. Duffey, Y. Jiang, D. Nguyen, A. Abakir, N. A. Willis, J. Houseley, R. Scully, Two-ended recombination at a Flp-nickase-broken replication fork. bioRxiv 2024.04.10.588130 [Preprint] (2024); https://doi.org/10.1101/2024.04.10.588130.

67. Y. Li, N. D. Roberts, J. A. Wala, O. Shapira, S. E. Schumacher, K. Kumar, E. Khurana, S. Waszak, J. O. Korbel, J. E. Haber, M. Imielinski, PCAWG Structural Variation Working Group, J. Weischenfeldt, R. Beroukhim, P. J. Campbell, PCAWG Consortium, Patterns of somatic structural variation in human cancer genomes. *Nature* **578**, 112–121 (2020). doi:10.1038/s41586-019-1913-9 Medline

68. S. Nik-Zainal, L. B. Alexandrov, D. C. Wedge, P. Van Loo, C. D. Greenman, K. Raine, D. Jones, J. Hinton, J. Marshall, L. A. Stebbings, A. Menzies, S. Martin, K. Leung, L. Chen,

C. Leroy, M. Ramakrishna, R. Rance, K. W. Lau, L. J. Mudie, I. Varela, D. J. McBride, G. R. Bignell, S. L. Cooke, A. Shlien, J. Gamble, I. Whitmore, M. Maddison, P. S. Tarpey, H. R. Davies, E. Papaemmanuil, P. J. Stephens, S. McLaren, A. P. Butler, J. W. Teague, G. Jönsson, J. E. Garber, D. Silver, P. Miron, A. Fatima, S. Boyault, A. Langerød, A. Tutt, J. W. M. Martens, S. A. J. R. Aparicio, Å. Borg, A. V. Salomon, G. Thomas, A.-L. Børresen-Dale, A. L. Richardson, M. S. Neuberger, P. A. Futreal, P. J. Campbell, M. R. Stratton, Breast Cancer Working Group of the International Cancer Genome Consortium, Mutational processes molding the genomes of 21 breast cancers. *Cell* **149**, 979–993 (2012). doi:10.1016/j.cell.2012.04.024 Medline

69. T. Hwang, S. Reh, Y. Dunbayev, Y. Zhong, Y. Takata, J. Shen, K. M. McBride, J. P. Murnane, J. Bhak, S. Lee, R. D. Wood, K. I. Takata, Defining the mutation signatures of DNA polymerase θ in cancer genomes. *NAR Cancer* **2**, zcaa017 (2020). doi:10.1093/narcan/zcaa017 Medline

70. A. Brambati, O. Sacco, S. Porcella, J. Heyza, M. Kareh, J. C. Schmidt, A. Sfeir, RHINO directs MMEJ to repair DNA breaks in mitosis. *Science* **381**, 653–660 (2023). doi:10.1126/science.adh3694 Medline

71. C. Gelot, M. T. Kovacs, S. Miron, E. Mylne, A. Haan, L. Boeffard-Dosierre, R. Ghouil, T. Popova, F. Dingli, D. Loew, J. Guirouilh-Barbat, E. Del Nery, S. Zinn-Justin, R. Ceccaldi, Polθ is phosphorylated by PLK1 to repair double-strand breaks in mitosis. *Nature* **621**, 415–422 (2023). doi:10.1038/s41586-023-06506-6 Medline

72. N. J. Panzarino, J. J. Krais, K. Cong, M. Peng, M. Mosqueda, S. U. Nayak, S. M. Bond, J. A. Calvo, M. B. Doshi, M. Bere, J. Ou, B. Deng, L. J. Zhu, N. Johnson, S. B. Cantor, Replication Gaps Underlie BRCA Deficiency and Therapy Response. *Cancer Res.* **81**, 1388–1397 (2021). doi:10.1158/0008-5472.CAN-20-1602 Medline

73. A. Quinet, S. Tirman, J. Jackson, S. Šviković, D. Lemaçon, D. Carvajal-Maldonado, D. González-Acosta, A. T. Vessoni, E. Cybulla, M. Wood, S. Tavis, L. F. Z. Batista, J. Méndez, J. E. Sale, A. Vindigni, PRIMPOL-Mediated Adaptive Response Suppresses Replication Fork Reversal in BRCA-Deficient Cells. *Mol. Cell* **77**, 461–474.e9 (2020). doi:10.1016/j.molcel.2019.10.008 Medline

74. A. Mehta, J. E. Haber, Sources of DNA double-strand breaks and models of recombinational DNA repair. *Cold Spring Harb. Perspect. Biol.* **6**, a016428 (2014). doi:10.1101/cshperspect.a016428 Medline

75. A. King, P. Reichl, J. S. Metson, R. Parker, D. Munro, C. Oliveira, J. R. Becker, D. Biggs, C. Preece, B. Davies, J. R. Chapman, Shieldin and CST co-orchestrate DNA polymerase-dependent tailed-end joining reactions independently of 53BP1-governed repair pathway choice. bioRxiv 2023.12.20.572534 [Preprint] (2023); https://doi.org/10.1101/2023.12.20.572534.

76. C. Doil, N. Mailand, S. Bekker-Jensen, P. Menard, D. H. Larsen, R. Pepperkok, J. Ellenberg, S. Panier, D. Durocher, J. Bartek, J. Lukas, C. Lukas, RNF168 binds and amplifies ubiquitin conjugates on damaged chromosomes to allow accumulation of repair proteins. *Cell* **136**, 435–446 (2009). doi:10.1016/j.cell.2008.12.041 Medline

77. G. S. Stewart, S. Panier, K. Townsend, A. K. Al-Hakim, N. K. Kolas, E. S. Miller, S. Nakada, J. Ylanko, S. Olivarius, M. Mendez, C. Oldreive, J. Wildenhain, A. Tagliaferro, L. Pelletier, N. Taubenheim, A. Durandy, P. J. Byrd, T. Stankovic, A. M. R. Taylor, D.

Durocher, The RIDDLE syndrome protein mediates a ubiquitin-dependent signaling cascade at sites of DNA damage. *Cell* **136**, 420–434 (2009). doi:10.1016/j.cell.2008.12.042 Medline

78. S. R. Wessel, K. N. Mohni, J. W. Luzwick, H. Dungrawala, D. Cortez, Functional Analysis of the Replication Fork Proteome Identifies BET Proteins as PCNA Regulators. *Cell Rep.* **28**, 3497–3509.e4 (2019). doi:10.1016/j.celrep.2019.08.051 Medline

79. A. J. Pierce, P. Hu, M. Han, N. Ellis, M. Jasin, Ku DNA end-binding protein modulates homologous repair of double-strand breaks in mammalian cells. *Genes Dev.* **15**, 3237–3242 (2001). doi:10.1101/gad.946401 Medline

80. J. Paiano, W. Wu, S. Yamada, N. Sciascia, E. Callen, A. Paola Cotrim, R. A. Deshpande, Y. Maman, A. Day, T. T. Paull, A. Nussenzweig, ATM and PRDM9 regulate SPO11-bound recombination intermediates during meiosis. *Nat. Commun.* **11**, 857 (2020). doi:10.1038/s41467-020-14654-w Medline

81. S. Yamada, A. G. Hinch, H. Kamido, Y. Zhang, W. Edelmann, S. Keeney, Molecular structures and mechanisms of DNA break processing in mouse meiosis. *Genes Dev.* **34**, 806–818 (2020). doi:10.1101/gad.336032.119 Medline

82. S. M. Noordermeer, S. Adam, D. Setiaputra, M. Barazas, S. J. Pettitt, A. K. Ling, M. Olivieri, A. Álvarez-Quilón, N. Moatti, M. Zimmermann, S. Annunziato, D. B. Krastev, F. Song, I. Brandsma, J. Frankum, R. Brough, A. Sherker, S. Landry, R. K. Szilard, M. M. Munro, A. McEwan, T. Goullet de Rugy, Z.-Y. Lin, T. Hart, J. Moffat, A.-C. Gingras, A. Martin, H. van Attikum, J. Jonkers, C. J. Lord, S. Rottenberg, D. Durocher, The shieldin complex mediates 53BP1-dependent DNA repair. *Nature* **560**, 117–121 (2018). doi:10.1038/s41586-018-0340-7 Medline

83. W. Wu, S. E. Hill, W. J. Nathan, J. Paiano, E. Callen, D. Wang, K. Shinoda, N. van Wietmarschen, J. M. Colón-Mercado, D. Zong, R. De Pace, H.-Y. Shih, S. Coon, M. Parsadanian, R. Pavani, H. Hanzlikova, S. Park, S. K. Jung, P. J. McHugh, A. Canela, C. Chen, R. Casellas, K. W. Caldecott, M. E. Ward, A. Nussenzweig, Neuronal enhancers are hotspots for DNA single-strand break repair. *Nature* **593**, 440–444 (2021). doi:10.1038/s41586-021-03468-5 Medline

84. G. Matos-Rodrigues, N. van Wietmarschen, W. Wu, V. Tripathi, N. C. Koussa, R. Pavani, W. J. Nathan, E. Callen, F. Belinky, A. Mohammed, M. Napierala, K. Usdin, A. Z. Ansari, S. M. Mirkin, A. Nussenzweig, S1-END-seq reveals DNA secondary structures in human cells. *Mol. Cell* **82**, 3538–3552.e5 (2022). doi:10.1016/j.molcel.2022.08.007 Medline

85. N. Wong, S. John, A. Nussenzweig, A. Canela, in *Homologous Recombination: Methods and Protocols*, A. Aguilera, A. Carreira, Eds., vol. 2153 of *Methods in Molecular Biology* (Humana Press, 2021), pp. 9–31.

86. J. Paiano, N. Zolnerowich, W. Wu, R. Pavani, C. Wang, H. Li, L. Zheng, B. Shen, B. P. Sleckman, B.-R. Chen, A. Nussenzweig, Role of 53BP1 in end protection and DNA synthesis at DNA breaks. *Genes Dev.* **35**, 1356–1367 (2021). doi:10.1101/gad.348667.121 Medline

87. E. Aronesty, Comparison of Sequencing Utility Programs. *Open Bioinform. J.* **7**, 1–8 (2013). doi:10.2174/1875036201307010001

88. M. Vasimuddin, S. Misra, H. Li, S. Aluru, “Efficient Architecture-Aware Acceleration of BWA-MEM for Multicore Systems” in *2019 IEEE International Parallel and Distributed Processing Symposium (IPDPS)* (IEEE, 2019), pp. 314–324.

89. J. L. Sparks, G. Chistol, A. O. Gao, M. Räschle, N. B. Larsen, M. Mann, J. P. Duxin, J. C. Walter, The CMG Helicase Bypasses DNA-Protein Cross-Links to Facilitate Their Repair. *Cell* **176**, 167–181.e21 (2019). doi:10.1016/j.cell.2018.10.053 Medline

90. D. Gómez-Cabello, G. Pappas, D. Aguilar-Morante, C. Dinant, J. Bartek, CtIP-dependent nascent RNA expression flanking DNA breaks guides the choice of DNA repair pathway. *Nat. Commun.* **13**, 5303 (2022). doi:10.1038/s41467-022-33027-z Medline

91. B. Langmead, C. Trapnell, M. Pop, S. L. Salzberg, Ultrafast and memory-efficient alignment of short DNA sequences to the human genome. *Genome Biol.* **10**, R25 (2009). doi:10.1186/gb-2009-10-3-r25 Medline

92. A. M. Bolger, M. Lohse, B. Usadel, Trimmomatic: A flexible trimmer for Illumina sequence data. *Bioinformatics* **30**, 2114–2120 (2014). doi:10.1093/bioinformatics/btu170 Medline

93. B. Langmead, S. L. Salzberg, Fast gapped-read alignment with Bowtie 2. *Nat. Methods* **9**, 357–359 (2012). doi:10.1038/nmeth.1923 Medline

94. H. Li, B. Handsaker, A. Wysoker, T. Fennell, J. Ruan, N. Homer, G. Marth, G. Abecasis, R. Durbin, 1000 Genome Project Data Processing Subgroup, The Sequence Alignment/Map format and SAMtools. *Bioinformatics* **25**, 2078–2079 (2009). doi:10.1093/bioinformatics/btp352 Medline

95. A. R. Quinlan, I. M. Hall, BEDTools: A flexible suite of utilities for comparing genomic features. *Bioinformatics* **26**, 841–842 (2010). doi:10.1093/bioinformatics/btq033 Medline

96. T. Smith, A. Heger, I. Sudbery, UMI-tools: Modeling sequencing errors in Unique Molecular Identifiers to improve quantification accuracy. *Genome Res.* **27**, 491–499 (2017). doi:10.1101/gr.209601.116 Medline

97. M. Martin, Cutadapt removes adapter sequences from high-throughput sequencing reads. *EMBnet. J.* **17**, 10–12 (2011). doi:10.14806/ej.17.1.200

98. Y. Zhang, T. Liu, C. A. Meyer, J. Eeckhoute, D. S. Johnson, B. E. Bernstein, C. Nusbaum, R. M. Myers, M. Brown, W. Li, X. S. Liu, Model-based analysis of ChIP-Seq (MACS). *Genome Biol.* **9**, R137 (2008). doi:10.1186/gb-2008-9-9-r137 Medline

99. W. J. Kent, C. W. Sugnet, T. S. Furey, K. M. Roskin, T. H. Pringle, A. M. Zahler, D. Haussler, The human genome browser at UCSC. *Genome Res.* **12**, 996–1006 (2002). doi:10.1101/gr.229102 Medline
